# Longitudinal Assessment of Twelve-Month Weight Loss Outcomes Post-Sleeve Gastrectomy: The Role of Serum and Fecal Metabolomic Biomarkers

**DOI:** 10.3390/metabo16070497

**Published:** 2026-07-15

**Authors:** Maya Nassif, Wendy M. Miller, Kathryn M. Ziegler, Nadia Ashrafi, Romana Mimi Ashrafi, Abdullah Khalid, Sumeyya Akyol, Jay Idler, Milda Milčiūtė, Vilija Lomeikaitė, Austėja Jankevičiūtė, Matthew D. Sims, Michael E. Maddens, Ali Yilmaz, Stewart F. Graham

**Affiliations:** 1 Oakland University William Beaumont School of Medicine, 586 Pioneer Dr, Rochester, MI 48309, USAali.yilmaz@corewellhealth.org (A.Y.); stewart.graham@corewellhealth.org (S.F.G.); 2 Corewell Health William Beaumont University Hospital, 3601 W. 13 Mile Road, Royal Oak, MI 48073, USA; 3 Corewell Health Research Institute, 3811 W 13 Mile Rd, Royal Oak, MI 48073, USA; 4 West Penn Hospital, Allegheny Health Network, 4815 Liberty Ave, Suite GR50, Pittsburgh, PA 15224, USA; 5 Drexel University College of Medicine, 2900 W Queen Ln, Philadelphia, PA 19129, USA; 6 VUGENE, LT-10312 Vilnius, Lithuaniaausteja@vugene.com (A.J.)

**Keywords:** metabolites, sleeve gastrectomy, weight loss, obesity, bariatric surgery, longitudinal metabolomics, biomarkers, machine learning

## Abstract

**Background/Objectives:** Metabolomics has emerged as a tool to gain insight into the body’s biological responses to therapeutic interventions. Bariatric surgery remains the most effective treatment for severe obesity and associated comorbidities, leading to significant weight and metabolic improvements. Using a multi-platform, multi-compartment metabolomics approach, this study systematically characterizes longitudinal changes in fecal and serum metabolomes of 45 patients following sleeve gastrectomy (SG). Participants were stratified by weight loss outcomes to identify metabolic signatures associated with differential responses, which may serve as predictors of weight loss and provide mechanistic insights in developing targeted therapeutic strategies. **Methods**: Metabolomic and lipidomic responses to SG were analyzed using multivariable linear mixed-effects models based on data collected pre-operatively and at 3 and 12 months post-operatively. **Results**: The percentage total weight loss for the highest versus lowest weight loss tertiles (T_3_ vs. T_1_) at twelve months was 35.81 + 5.4% and 19.49 + 2.62%, *p* < 0.001, respectively. Substantial alterations in fecal metabolites and lipid species were observed among T3 after twelve months, including aspartate, tyrosine, carnitine, glycine, PC.ae.C36.4, PC.aa.C38.0, PC.ae.C44.4, PC.ae.C40.2, and PC.aa.C40:5. Specifically, changes in serum lipid species including SM.OH.C22:1, PC.ae.C32:1, PC.aa.C34:4, PC.aa.C36:6, PC.ae.C34:3, PC.ae.C34:1, and PC.ae.C32:2, support serum lipidomics as a minimally invasive marker of gut remodeling and adaptation following SG. Sex-stratified analysis revealed unique fecal and serum metabolic changes, highlighting the significance of personalized metabolic monitoring and obesity treatment. **Conclusions**: Our findings identify metabolic alterations associated with response variability following SG and highlight the potential utility of machine learning to predict weight-loss trajectories and inform personalized interventions.

## 1. Introduction

Obesity has been increasing at an alarming rate since the 1980s and continues to cement its significance as one of the world’s most prominent public health issues with no expectation of slowing down. In a study projecting future prevalence of obesity using national body mass index (BMI) distribution data, it is anticipated that 85% of adults in the United States will be overweight or obese (BMI ≥ 25.0), with 50% having obesity (BMI ≥ 30.0) by 2030 [1]. The complexity of obesity is underscored by its numerous sequelae and comorbidities, including hypertension, cardiovascular disease, and diabetes, as well as the substantial economic burden on the healthcare system, with some estimates exceeding $215 billion annually [2,3].

Traditional obesity control measures such as diet and exercise fail for several reasons, including poor adherence, lack of sustainability, genetic predisposition and hormonal changes, leaving room for bariatric procedural options like sleeve gastrectomy (SG) in patients who cannot maintain a healthy weight [4,5]. In the last several years, SG has become the most commonly performed bariatric surgery in the United States, replacing Roux-en-Y gastric bypass (RYGB), due to its fewer complications and reduced invasiveness [6]. The definition of success or failure post-bariatric surgery can be nuanced for each clinician, and thus is more systematically assessed using the Bariatric Analysis and Reporting Outcome System (BAROS) [7]. A patient’s BAROS score can classify their results into one of five outcome groups: failure, fair, good, very good, or excellent [7]. What predicates a patient’s classification involves several intertwining elements. Suboptimal weight loss is noted to be associated with older age, recurrence of obesity-related comorbidities, depression/anxiety and social factors like unemployment, while success is correlated with lower preoperative BMI, younger age, and female gender [8]. Additionally, anthropometric measures that capture central adiposity offer important context for assessing obesity-related metabolic risk, given their strong association with cardiometabolic comorbidities [9].

In conjunction with the aforementioned factors that influence post-SG weight loss, metabolomics has proven itself to be an underutilized approach for predicting outcomes and developing therapeutic targets to enhance weight loss after SG. Furthermore, metabolomic and lipidomic profiling can help elucidate the molecular mechanisms driving metabolic improvements following bariatric surgery. Using a combination of proton nuclear magnetic resonance (^1^H NMR) spectrometry and targeted mass spectrometry, our group and others have successfully employed metabolomics-based profiling to assess metabolic signatures across a range of interventions, including dietary modification, physical activity, and SG [10,11,12].

The growing interest in recent years surrounding the gut microbiome lends itself to the incorporation of fecal metabolomic data to better understand health and disease. Although serum metabolites remain significant in the analysis of systemic metabolism and organ function, fecal metabolites are useful in that their derivation from the gut offers a unique lens when interpreting the complexities of obesity [13]. Cui et al. [14] evaluated the correlation between the human fecal metabolome and BMI, finding that subjects with a BMI ≥ 27 showed higher levels of fecal valine, alanine and phenylalanine than subjects with a BMI ≤ 23.5. We believe that integrating both serum and fecal metabolomic data offers the ability to better understand obesity and the effects the gut microbiome-metabolome has on bariatric surgery outcomes.

Maximal BMI changes post-SG typically occur between one and two years post-surgery, with most patients experiencing the greatest decrease in BMI and weight within the first year [15,16]. While early postoperative weight loss has been suggested to predict later weight outcomes, variability in the timing of weight and BMI decline highlights the importance of long-term patient follow-up and metabolomic investigation beyond three months [17].

Building on our previous work, which examined weight loss outcomes and their association with serum and fecal metabolomic data, the present study extends this analysis to longer-term outcomes. In our prior study, patients in the highest weight loss tertile demonstrated distinct serum and fecal metabolite alterations. Additionally, preoperative metabolite profiles showed strong predictive performance for postoperative weight loss outcomes using machine learning approaches. Similar to our initial three-month follow-up study, we hypothesize that there is a direct association between postoperative serum and fecal metabolomic profiles and weight loss outcomes [12]. Further, we hypothesize that preoperative metabolomic profiles may help inform future prognostic models of twelve-month weight loss outcomes following SG.

## 2. Materials and Methods

### 2.1. Clinical Cohort

This prospective single-arm observational study enrolled 45 adults with obesity who participated in a bariatric surgery program at Corewell Health East William Beaumont University Hospital (Royal Oak, MI, USA) between February 2018 and April 2019. All participants were scheduled to undergo laparoscopic sleeve gastrectomy (SG). The present cohort represents an interim analysis of an ongoing longitudinal study designed to include 150 patients, with postoperative follow-up assessments conducted at 3 and 12 months, respectively. Metabolomic profiling was performed on paired fecal and serum samples collected at 3 months (*n* = 45) and 12 months (*n* = 37) post-SG. Participants were stratified into tertiles according to the percentage of total weight loss (%TBWL) achieved postoperatively. At 3 months following SG, each tertile comprised 15 participants. At 12 months, the distribution included 13 participants in the first tertile (T_1_), 10 in the second tertile (T_2_), and 14 in the third tertile (T_3_).

The study protocol was reviewed and approved by the Corewell Health Institutional Review Board (nee Beaumont Health; IRB# 2017-201), and all procedures were conducted in accordance with institutional and ethical guidelines.

Eligible participants were adults aged 18 to 70 years with a BMI ≥ 40 kg/m^2^, or a BMI between 35 and 40 kg/m^2^ in the presence of at least one obesity-related comorbidity, including type 2 diabetes mellitus, cardiovascular disease, or obstructive sleep apnea. All participants were enrolled in the health system’s multidisciplinary bariatric surgery program. Individuals were excluded if they were unable to comply with scheduled postoperative follow-up visits, were pregnant at the time of enrollment, or had any medical or psychiatric condition that, in the opinion of the investigator, could interfere with their ability to participate fully and adhere to study procedures.

### 2.2. Measures and Samples

Serum and fecal samples were collected at preoperative, three-month, and twelve-month postoperative time points. Fecal samples were self-collected by participants at home using a stool collection kit at both three months and twelve months post-SG. After collection, fecal samples were kept in a home freezer for 24 to 48 h before being transferred on ice in an insulated bag to the research staff. Preoperative samples were taken before the start of the calorie-restricted preoperative diet. Both serum and fecal samples were then preserved at −80 °C in the biorepository within the Corewell Health Research Institute. Demographic information, such as weight, height, and BMI, was documented at the time of enrollment in the bariatric surgery program. Weight was measured again on the day of SG surgery, and subsequently at three months and twelve months postoperatively. Participants were grouped into tertiles based on the percentage of total weight loss (%TWL) at three- and twelve-months post-SG, calculated as follows: (%TWL = [preoperative weight − weight at three, or twelve months postoperatively]/preoperative weight). T3 includes individuals with the highest %TWL, while T1 includes those with the lowest %TWL.

### 2.3. Metabolomic Analyses

#### 2.3.1. ^1^H NMR Analysis Sample Preparation

##### Fecal Samples

Frozen stool samples were thawed on ice, and 65 (±5) mg aliquots were transferred into microcentrifuge tubes. 600 µL of cold deionized water (4 °C) was added to each sample. The samples were vortexed for 10 min, followed by shaking at 200 rpm for 30 min at 4 °C. The resulting mixtures were then centrifuged at 15,000× *g* for 30 min at 4 °C. Subsequently, 500 µL of the supernatant was transferred to 3 KDa cut-off centrifugal filter units (Amicon Microcon YM-3; Sigma-Aldrich, St. Louis, MO, USA) and centrifuged at 13,000× *g* for 30 min at 4 °C. A 200 µL aliquot of the resulting extract was then mixed with 25 µL of deuterated water (D_2_O) and 21 µL of a standard buffer solution containing 11.7 mM DSS-d_6_ (disodium-2,2-dimethyl-2-silapentane-5-sulfonate), 1.75 M K_2_HPO_4_, and 5.84 mM 2-chloro pyrimidine-5-carboxylic acid (phasing standard) in H_2_O, with 0.1% w/v sodium azide. The pH of the mixture was measured and, if necessary, adjusted to 7.4, after which the sample underwent an additional centrifugation step. Finally, the resulting mixture was transferred into a 3 mm high-resolution NMR tube (Wilmad, Buena, NJ, USA) for ^1^H NMR spectroscopic analysis.

##### Serum Samples

Serum samples were prepared using a modified protocol based on the method outlined by Mercier et al. [18]. In brief, 3 KDa cut-off centrifugal filter units (Amicon Microcon YM-3; Sigma-Aldrich, St. Louis, MO, USA) were rinsed seven times by centrifugation (12,000× *g* for 20 min) using 0.5 mL of H_2_O to remove any residual glycerol. Subsequently, 300 μL of serum was transferred to the filter units and centrifuged at 13,000× *g* for 30 min at 4 °C. To 228 µL of the filtrate, 28 µL of D_2_O and 24 µL of 11.77 mM DSS-d_6_ in 50-mmol NaH_2_PO4 buffer (pH 7) were added. Using a liquid handler system (Bruker Biospin, Billerica, MA, USA), 200 µL of the mixture was then transferred to a 3 mm NMR tube for analysis by NMR.

##### ^1^H NMR Data Acquisition

All 1D ^1^H NMR spectra were obtained on a Bruker ASCEND III 600 MHz spectrometer (Bruker Biospin, Billerica, MA, USA) equipped with a triple resonance 5 mm Cryo-Probe. Samples were automatically loaded into the spectrometer using a SampleJet sample changer (Bruker, Billerica, MA, USA). The samples were stored between 5–10 °C and each sample was prewarmed to 25 °C just before insertion into the spectrometer. NMR spectra for fecal metabolomics analysis were acquired using a modified version of a 1D ^1^H NOESY pulse sequence (noesygppr1d) with a 2 s pre-saturation pulse for water suppression, a 10 ms mixing time with water saturation, and a 4.56 s acquisition time [19]. Each sample was run for 256 scans to a total acquisition size of 128 k, a spectral window of 20.5 ppm, and a transmitter offset of ≈4.7 ppm. In addition, the transmitter offset was optimized to ensure optimal water suppression before the start of data collection on each sample, hence the reported offset of ≈4.7 ppm. Before ^1^H NMR data acquisition, three-dimensional and one-dimensional shimming experiments were conducted on the samples to correct any inhomogeneities in the static magnetic field. Spectra with poor line shape qualities or linewidths greater than 1 Hz were re-acquired. For each sample, spectral processing was carried out using Chenomx (Chenomx Inc., Edmonton, AB, Canada); the 1D version of the NOESY spectra was processed with exponential line broadening such that the DSS peak width was 1 Hz. In all cases, the manual phase and baseline correction Whittaker Spline function was applied. For manual profiling, fecal samples were quantified using the Chenomx NMR Suite version 9.1 using a combination of the software-provided 600 MHz compound library and an in-house compound library acquired at 600 MHz.

#### 2.3.2. Targeted Metabolomics of Feces and Serum Using Liquid Chromatography-

##### Mass Spectrometry (LC-MS)

LC-MS grade acetonitrile, methanol, isopropyl alcohol, and formic acid (≥99.0% purity) were obtained from Fisher Scientific (Hanover Park, IL, USA), while LC-MS grade ethanol, pyridine, and phenylisothiocyanate were purchased from Sigma Aldrich (St. Louis, MO, USA), with Milli-Q water (EMD Millipore, Billerica, MA, USA) used for the aqueous mobile phase. Lyophilized fecal samples (50 mg) were extracted using ethanol/phosphate buffer (85:15) and serum per biocrates’ instructions. Extracts were mixed, centrifuged (10,000× *g* at 4 °C for 10 min), and calibration standards and quality controls (QCs) were prepared in H_2_O. Samples (fecal, serum, standards, QCs) were added to a 96-well plate, dried under nitrogen for 30 min, derivatized with PITC for 60 min, and dried under nitrogen for 60 min. Extracts were obtained in 5 mM ammonium acetate in methanol for 30 min, centrifuged at 500× *g* for 2 min, and diluted with H_2_O (1:1) for LC analysis. For flow injection analysis (FIA), 50 µL of extract was mixed with 450 µL of kit solvent. LC and FIA plates were sealed, mixed at 600 rpm for 10 min, and loaded into an autosampler for analysis. Fecal extracts were analyzed using an Acquity Ultra-Performance Liquid Chromatography (UPLC)-I class coupled with a Waters Xevo-TQ-S (Waters Corporation, Milford, MA, USA). Separation was performed on an MxP Quant 500 C18 column (biocrates, Innsbruck, Austria) with a mobile phase of H_2_O/formic acid (0.2%) (A) and MeCN/formic acid (0.2%) (B) at 0.8 mL/min, following a 0–100% B gradient over 4.5 min. The flow rate increased to 1.0 mL/min for 30 s, then returned to initial conditions with a 70 s equilibration. The 5.8 min gradient varied between positive and negative modes (2.0–4.5 min %B difference). Injection volumes were 5 µL (positive) and 15 µL (negative), with a wash solvent of H_2_O: MeOH: MeCN: IPA (*v*/*v*). The Q500 kit enabled direct flow injection (FIA) for lipid analysis using an isocratic method with the kit solvent (290 mL MeOH + FIA additives). The mobile phase (100% MeOH) was delivered at 0.03 mL/min, with a 20 µL injection volume for both positive and negative modes. Three QC samples at low, mid, and high concentrations (biocrates, Innsbruck, Austria) were used, and data extraction was done using biocrates’ MetIDQ software (MetIDQ-Oxygen-DB110-3005).

### 2.4. Statistical Analyses

The metabolomics data were normalized sample-wise using sample quantile normalization, a powerful technique that aligns the statistical distribution of metabolite concentrations across different samples. By standardizing the distribution of metabolites, this method effectively minimizes technical variability, such as differences in sample preparation or instrument response, which could otherwise introduce bias into the analysis. As the longitudinal metabolomics data were acquired across two analytical batches, potential batch effects were addressed using ComBat-Seq function in the Bioconductor sva package v3.48.0 in R v3.17, which adjusts the metabolomics data to account for differences in sample preparation and instrumentation [20]. This method applies empirical Bayesian linear modeling to stabilize variance and improve statistical power in the data. The variation due to the project was deemed to be an unwanted variation and was removed.

Consequently, normalization ensures that the observed differences in metabolite levels between samples are more likely to reflect true biological variation rather than artifacts introduced during data collection or processing. Metabolites with zero variance (i.e., those whose abundances did not differ between samples) were dropped. Then, metabolites with more than 30% of missing values among all samples were removed from further analysis. Any remaining missing values were imputed using the R package impute v1.76.0 using the K-nearest neighbor (K-NN) algorithm and 5 nearest neighbors.

In order to detect outlier samples, principal component analysis (PCA) of filtered and log-transformed concentration values was computed, and samples with higher than 3 standard deviations (SDs) in the first 3 principal components (PCs) were marked as outliers. The detected outliers were removed from further analysis.

#### 2.4.1. Linear Modeling

Any metabolite that had >20% missing values in any group was excluded from further analysis. Normalized metabolite concentration values were used to fit the linear regression model using the R package limma v3.56.2 [21]. The model was adjusted for Condition, Sex and Age. Adjustment for unknown confounders was performed using the R package RUVSeq v1.34.0, which works by first identifying metabolites not related to the condition of interest and using them as a control set. The algorithm then estimates unwanted variability, and those estimates are passed into subsequent linear modeling as covariates. By identifying and adjusting for these sources of technical variation, RUVSeq helps improve the accuracy and reliability of downstream analyses. RUVg vector was used. Before fitting the model, duplicate correlation was evaluated using the duplicate Correlation function from the R package limma v3.56.2. The models were fitted using the least-squares method by running the lmFit function. This method minimizes the sum of the squared differences between observed values and the values predicted by the model, providing estimates for the model parameters. Empirical Bayes statistics were estimated using the eBayes function. The Benjamini–Hochberg (BH) method was used to correct for multiple testing. Correction for multiple testing is needed to control the false discovery rate (FDR) when conducting multiple statistical tests simultaneously. Without correction, the probability of observing false positives increases with the number of tests performed, leading to an inflated rate of false discoveries. Correcting for multiple testing helps maintain the overall error rate at a desired level, ensuring that significant findings are more likely to represent true associations rather than chance findings. Metabolite concentration differences with q-value < 0.05 were deemed significant.

The difference here is the effect of the variable of interest. Several pairwise comparisons have been carried out in both cross-sectional and longitudinal manners. These include baseline (BL) vs. 12-month Tertile1 (12M_T_1_), BL vs. 12-month Tertile2 (12M_T_2_), and BL vs. 12-month Tertile3 (12M_T_3_). Further, statistical analysis using linear modeling on data also stratified by sex to compare different groups while accounting for covariates, thereby enabling identification of sex-specific effects and enhancing biological interpretation within a personalized medicine framework.

#### 2.4.2. Metabolites Functional Analysis

Before machine learning model development, sample identifiers were selected from the normalized dataset for each group. Metabolites were filtered by variance, retaining the top 30 most variable metabolites. Pairwise Pearson correlations were calculated using complete observations. Correlation matrices were visualized as circular heatmaps with hierarchical clustering, where colors indicate correlation direction and circle size reflects strength, using the corrplot R package v0.95. This analysis was performed separately for each group.

In the current study, machine learning algorithms, GLMnet and svmRadial, were utilized to classify weight-loss response groups at twelve months post-SG using caret R package v7.0.1. We have trained pairwise classifiers that discriminate between baseline (BL) and 12 months after surgery tertile groups (M12_T_1_, M12_T_2_, M12_T_3_), as well as 3 months after surgery (M3).

In order to obtain estimates of classifier performance, we created 10 different data partitions: 10 different training and validation datasets were created, with 70% of the samples in the training subset and 30% of the samples in the validation subset. Partitioning was done at the participant level to account for the repeated measures by ensuring that all longitudinal samples (baseline and postoperative) from any given individual are strictly kept together within the same subset. For each of the 10 cross-validation iterations, three machine learning algorithms were trained and evaluated:

GLMnet: A generalized linear model with elastic net regularization, which combines L1 (Lasso) and L2 (Ridge) penalties.

Support Vector Machine with Radial Basis Function kernel (SVM-R): A kernel-based method that finds an optimal hyperplane to separate classes in a high-dimensional space.

For each algorithm, hyperparameter tuning was performed within the training folds using repeated 7-fold cross-validation repeated 5 times. The tuning process explored 15 different hyperparameter combinations for each model. BL versus M12_T_3_ model underwent 6-fold cross-validation (also repeated 5 times with 15 tuning hyperparameter combinations). This was necessary because the BL vs. 12M_T_3_ group only had nine samples overall, of which six were in the training set (7-fold CV is not possible). The performance of the models during training was evaluated using the Mean Balanced Accuracy, calculated using a custom metrics calculation function, which provides a comprehensive set of classification metrics (ROC, Specificity, Sensitivity). We then used the validation dataset of each partition to evaluate the performance of the best classifier on data it had not seen yet. Following the cross-validation procedure, a final set of the same machine learning algorithms (GLMnet and SVM-R) was trained on the entire dataset using the same hyperparameter tuning strategy (repeated 7-fold cross-validation repeated 5 times, tuneLength = 15) and evaluation metric (Mean Balanced Accuracy) as described above.

The random forest model was not used on this data because the data in subgroups is fairly unbalanced (most samples belong to the BL category). The random forest model has an inherent bias towards the majority class, thus predicting all of the samples as BL, giving rise to inaccurate prediction metrics due to the high true positive (TP) rate of predictions and a low number of samples from the negative class.

### 2.5. Metabolites Functional Analysis

To further investigate the metabolic processes involved in weight loss, metabolite functional analysis was conducted as follows: normalized metabolite concentrations were grouped according to their functional categories, and statistical evaluation using a mixed-effects model was performed for each metabolite class. Metabolites within each category were reshaped and combined with metadata relevant to the covariates in the linear model. Metabolite names were treated as variables, with their corresponding concentrations used as values. Subsequently, we used the limma R package v3.56.2 to conduct the analysis. The results of functional categories metabolite analysis using a linear mixed-effects model are plotted using a dot plot. The *x*-axis labels the metabolite’s biological functions. On the *y*-axis, each dot represents an estimate, while whiskers show the standard errors. An estimate is the expected change in the dependent variable for a one-unit increase in the corresponding covariate, assuming all other covariates are held constant. For categorical variables (like Condition), the estimate shows the difference in the response between the given category (e.g., “Case”) and the reference category (e.g., “Control”). For example, if estimates for one of the contrasts equal 0.5 for a certain biological function, it means that, on average, case subjects versus controls have 0.5 units higher concentration value for that biological function, assuming all other variables in the model are held constant.

## 3. Results

Baseline characteristics of the patient cohort and the highest weight loss tertile (T3) versus the lowest weight loss tertile (T_1_) are shown in Table 1. The average %TWL at twelve months post-SG was 27.25 + 7.66%. The %TWL for T_3_ versus T_1_ at twelve months post-SG was 35.81 + 5.4% and 19.49 + 2.62%, *p* < 0.001.

### 3.1. Quality Checking of the Metabolomics Data

Using PCA, we first sought to determine if there were any systematic variations or outliers in our dataset. PCA identified four outliers in the fecal metabolomics dataset, while no outliers were detected in the serum metabolomics dataset. A substantial batch effect was evident in the score plots before data processing; however, PCA performed after batch correction showed effective removal of batch-associated clustering, indicating robust normalization of both metabolomics datasets. (Supplementary Figures S1a,b and S2a,b) To better understand the variation structure in the dataset, variation across the principal components was investigated. In the fecal metabolomics dataset, PC1 and PC2 accounted for 9.8% and 8.54% of the total variance, respectively. Although these principal components captured the largest proportion of variance in the dataset, the score plots showed no clear separation between the control, 3M, and 12M groups. On the other hand, in the serum metabolomics dataset, PC1 and PC2 accounted for 13.41% and 11.92% of the total variance, respectively, with score plots demonstrating distinct separation among the sample groups. Histogram graphs displayed the overall signal intensity distribution across both metabolomic datasets after preprocessing, with aggregated conditions. Overlaid group-specific density plots showed uniform and well-normalized signal profiles, indicating minimal technical variation and confirming data suitability for subsequent statistical and bioinformatic analyses (Supplementary Figures S3A,B and S4A,B).

### 3.2. Linear Modeling

**Figure 1:**
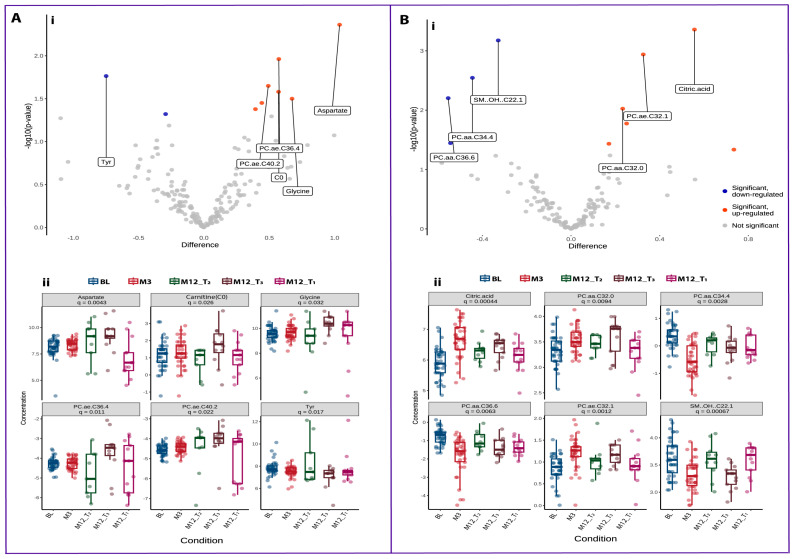
(**A**) Volcano plot comparing fecal metabolic profile of patients at BL with fecal metabolic profile of super responsive patients at post operative 12 month (**i**) the box plots showing concentration of the top six fecal metabolites revealed by linear models revealing for this comparison and corresponding longitudinal changes at BL, 3M and 12M at different tertiles (**ii**) and (**B**) Volcano plot comparing serum metabolic profiles of patients at BL with fecal metabolic profile of super responsive patients at post operative 12 month (**i**) the box plots showing concentration of the top six fecal metabolites revealed by linear models revealing for this comparison and corresponding longitudinal changes at BL, 3M and 12M at different tertiles (**ii**).

**Figure 2:**
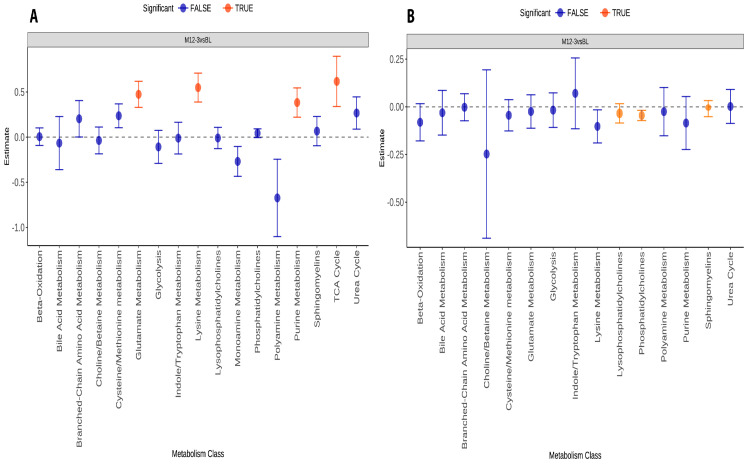
Results of the functional metabolite analysis based on fecal metabolomics data for comparison of BL with 12M_T_3_ (**A**) and result of the same comparison when using serum metabolomics data (**B**). Blue indicates pathways showing no significant perturbations and orange indicates metabolic pathways showing statistically significant perturbations.

Before linear modeling, a correlation heatmap was generated to visualize relationships among covariates and the first five principal components. Sex and age were identified as confounding factors influencing the serum metabolomic profile, whereas only age was a significant confounder in the fecal metabolomic profile (Supplementary Figures S5 and S6). To identify metabolites showing significant concentration changes, we fitted a least squares linear regression model that accounted for possible unknown sources of unwanted variation and potential confounders such as sex and age. Linear models based on fecal metabolomics data revealed that 9 out of 49 quantified metabolites showed statistically significant concentration changes when comparing BL with 12M_T_3_ groups (Figure 1A, Supplementary Table S1). Within this comparison, the most significantly affected metabolites were found to be aspartate, tyrosine, carnitine, glycine, PC.ae.C36:4, PC.aa.C38:0, PC.ae.C44.4, PC.ae.C40:2, and PC.aa.C40.5 (*p*-value < 0.05) (Figure 1B). Similarly, linear models comparing serum metabolic profile differences between the BL and 12M_T_3_ groups identified SM.OH.C22:1, PC.ae.C32:1, PC.aa.C34:4, PC.aa.C36:6, PC.ae.C34:3, PC.ae.C34:1, and PC.ae.C32:2 as metabolites with statistically significant concentration changes (*p*-value < 0.05) (Figure 2A,B, Supplementary Table S2). Furthermore, for the same pairwise comparisons, sex-stratified linear models were applied to metabolomics data to identify sex-specific metabolic signatures in both fecal and serum samples following SG. Remarkably, volcano plots from the sex-stratified analyses revealed substantial sex-associated differences in both fecal and serum metabolomes (Supplementary Figure S7A(i,ii),B(i,ii)). Similarly, marked metabolic shifts were observed among T_1_ and T_2_ at 12M (12M_T_1_ and 12M_T_2_) when compared with corresponding BL fecal and serum metabolomics data.

### 3.3. Metabolites Functional Analysis

Functional pathway analysis based on the fecal metabolomics dataset yielded a limited number of statistically significant alterations, likely reflecting the relatively small sample size and corresponding constraints in statistical power. Among the metabolic pathways examined, lysine metabolism was the only pathway that demonstrated a statistically significant upregulation when the BL group was compared with the 12M_T_3_ group (Figure 2A). Although remaining pathways did not reach statistical significance after correction, it is noteworthy that several metabolic pathways exhibited divergent regulatory patterns depending on the response phenotype. Specifically, when non-responders and super-responders were evaluated relative to baseline, certain pathways, including the TCA cycle, bile acid metabolism, lysophosphatidylcholine metabolism, beta-oxidation, branched-chain amino acid metabolism, and lysine metabolism, showed opposite directional changes (upregulation versus downregulation).

A similar functional analysis performed using the serum metabolomics dataset revealed significant perturbations in several lipid metabolism-related pathways following SG. In particular, metabolic pathways associated with sphingomyelin metabolism, phosphatidylcholine metabolism, and lysophosphatidylcholine metabolism exhibited significant alterations (*p*-value < 0.005) (Figure 2B).

To gain deeper insight into the metabolic responses associated with weight loss following SG, a sex-stratified functional metabolite analysis was also performed separately for both fecal and serum metabolomics datasets. This approach enabled the evaluation of sex-specific metabolic alterations and facilitated the identification of pathways that may differentially contribute to the metabolic response to SG in men and women. Notably, this analysis revealed significant alterations in several metabolic pathways that were unique to each sex, indicating distinct metabolic remodeling patterns in males and females across both fecal and serum metabolomic profiles (Supplementary Tables S3 and S4).

While the sample size limits us from drawing a general conclusion, they may nonetheless reflect biologically meaningful metabolic differences associated with treatment response and warrant further investigation in studies with larger cohorts and greater statistical power. For serum metabolomics data, substantial remodeling of circulating lipid metabolites after the surgical intervention was indicated, highlighting that SG induces pronounced changes in systemic lipid metabolic processes.

### 3.4. Classification Modeling

Before building machine learning classification models, the correlation structures of the top 30 most influential metabolites in each group were investigated (Supplementary Figure S8A,B). Substantial differences in inter-metabolite covariation structure across groups reflected distinct metabolic network organization, underscoring the potential for machine learning-based predictive modelling to capture these group-specific signatures. Moreover, characteristic metabolic structural differences among the three groups (BL, 3M and 12M) in both fecal and serum metabolomic signatures highlight the longitudinal changes occurring after SG.

**Figure 3:**
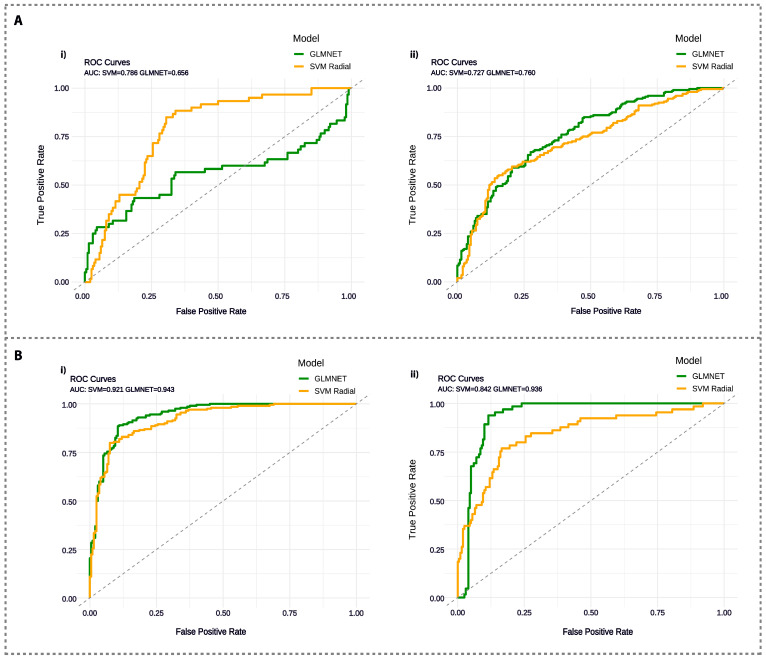
(**A**) Fecal metabolite classification models and receiver-operating characteristic (ROC) plots for twelve-month post-sleeve gastrectomy weight loss outcomes: Highest %TWL tertile (T3) versus all patients at baseline (**i**) and for three-month post-sleeve gastrectomy weight loss outcomes: Highest %TWL tertile (T3) versus all patients at baseline (**ii**) and (**B**) Serum metabolite predictive models and receiver-operating characteristic (ROC) plots for twelve-month post-sleeve gastrectomy weight loss outcomes: Highest %TWL tertile (T3) versus all patients at baseline (**i**) and for three-month post-sleeve gastrectomy weight loss outcomes: Highest %TWL tertile (T3) versus all patients at baseline (**ii**).

In the BL vs. M12_T_3_ comparison, classification modeling based on twelve-month postoperative fecal metabolomics data yielded moderate discriminatory performance. The GLMNET classification model achieved an AUC of 0.78 and a mean balanced accuracy of 0.72, while the radial SVM model demonstrated comparatively lower performance, with an AUC of 0.74 and a mean balanced accuracy of 0.65 (Figure 3A). Within this comparison, the top five most influential fecal metabolites by Recursive Feature Elimination (RFE) were found to be PC.aa.C40.1, Tyr, PC.ae.C42.3 and lysoPC.a.C24.0 for GLMNET and PC.ae.C40.2, PC.ae.C36.4, PC.aa.C38.0, PC.aa.C40.3 and PC.ae.C38.5 for SVM Radial models. Similarly, for the same group comparison, classification models based on serum metabolomics data at the twelve-month postoperative time point demonstrated superior predictive power. The GLMNET model achieved an AUC of 0.90 with a mean balanced accuracy of 0.90, while radial SVM approaches yielded comparable discriminatory ability, providing an AUC of 0.86 and a mean balanced accuracy of 0.80. (Figure 3B). Using RFE, serum metabolomics models identified the five most influential metabolites as SM.OH.C22:1, citrate, PC.aa.C36.6, creatine, and PC.aa.C32.0 for GLMNET and lactate, acetone, 3-hydroxybutyric acid, PC.aa.C36:6, and PC.aa.C34.3 for SVM Radial models.

GLMNET and SVM Radial models based on fecal metabolomics data at baseline and three months post-operatively (BL vs. M12_T3) provided discriminatory performance with AUCs of 0.80 and 0.74 and mean balanced accuracy of 0.74 and 0.70, respectively. In these models, the top five metabolites for GLMNET, as selected by RFE, were PC.aa.C40:4, PC.aa.C42:0, PC.ae.C40:3, PC.ae.C36:4, whereas the top five metabolites for the SVM Radial model were lactic acid, PC.aa.C36:6, citrate, PC.aa.C34:4 and PC.ae.C32:1. For the same group comparison, serum-based classification models demonstrated superior performance. The GLMNET approach achieved an AUC of 0.95 with a mean balanced accuracy of 0.86, while the radial SVM model showed comparable performance, with an AUC of 0.92 and a mean balanced accuracy of 0.85. In these models, RFE identified SM.C24:1, PC.aa.C36:5, PC.aa.C34:4, SM.OH.C16:1, and creatine as the top five metabolites distinguishing the three-month post-operative cases from baseline patients. Given the modest sample size, this machine learning classification should be interpreted as exploratory. Although cross-validation was used to estimate model performance, the reported AUC values may be optimistic and require validation in larger, independent cohorts.

## 4. Discussion

SG remains among the most effective interventions for achieving sustained weight reduction and improvement in comorbidities in patients with severe obesity. Contemporary obesity management is increasingly multimodal, spanning lifestyle changes, pharmacotherapy, and bariatric surgery. The rising visibility and uptake of incretin-based medications (GLP-1 receptor agonists) reflects their substantial weight loss efficacy; however, evidence demonstrates meaningful weight regain and even reversal of beneficial effects on cardiometabolic markers after medication withdrawal [22]. Thus, maintaining the benefits of such medications typically requires continued treatment, and combined with gastrointestinal adverse effects, poses clear practical limitations. In contrast, SG has been associated with more and sustained weight loss at lower ongoing costs in class II and III obesity, reinforcing the continued clinical relevance and efficacy of SG in the new era of anti-obesity medications [23]. As such, systematic characterization of the molecular response to surgical weight loss is essential to improving clinical management and facilitating personalized therapeutic strategies. By extending our previous 3-month analysis further into the postoperative phase, this work notably provides a longitudinal pathway-level view of metabolic remodeling and its association with weight loss success post-SG.

To our knowledge, this is the first and most comprehensive multi-tissue metabolomic assessment evaluating metabolite and lipid changes over time, capturing both short-term changes at 3 months post-surgery and longer-term changes at 12 months, as well as associations with weight loss success following SG, enabled in part by our integrated, multi-platform approach and further strengthened by sex-stratified analyses. Although there is some degree of overlap, each metabolomic platform identifies a substantially unique class of metabolites. A combination of platforms, therefore, yields significantly more information on the diverse biochemical processes involved in the biological response to SG. Therefore, unlike the conventional approach, which relies on only a single analytical platform, we combined complementary 1D ^1^H NMR spectroscopy and MS-based metabolomics to generate more comprehensive metabolic profiles so that one can corroborate key metabolic signatures associated with weight loss following SG. Moreover, the present study is further strengthened by its multi-tissue profiling approach, integrating metabolic characterization of both serum and fecal metabolomes.

Because most bariatric metabolomics studies prioritize serum-based signatures, inclusion of fecal profiling offers a unique lens into gut-associated processes that may be less apparent in systemic circulation [24,25]. Additionally, much of the existing literature characterizes metabolic changes after SG not longitudinally but rather at a single postoperative time point, making it difficult to distinguish transient biochemical changes from sustained metabolic remodeling linked to long-term weight loss [24]. By evaluating patients at both 3 and 12 months, our longitudinal design captures between-patient differences in metabolomic profiles over time and clarifies how such patterns align with durable weight loss outcomes. This distinction is particularly relevant after SG, where rapid early physiological changes may not predict sustained weight loss.

To identify metabolites with significantly different abundances between stratified groups of samples, while accommodating multiple covariates, adjusting for known and unknown confounding factors such as age and gender was conducted. Notably, linear models revealed that concentrations of several metabolites were perturbed in patients 12 months post-SG, many of which exhibited tertile-specific differences associated with the degree of weight loss.

Among patients in the highest weight loss group (T_3_), linear modeling of fecal metabolomic data identified 10 significantly altered metabolites, 7 of which were lipid-associated species, including phosphatidylcholines and acylcarnitines. The remaining three were amino acids aspartate, tyrosine, and glycine, suggesting concurrent alterations in amino acid metabolism. Amino acids and related metabolites comprised one of the largest classes of metabolites altered following bariatric surgery, consistent with findings from previous studies [24,26,27]. Specifically, the predominance of phosphatidylcholines (phospholipid metabolism) and acylcarnitines (fatty acid trafficking and β-oxidation) is consistent with shifts in lipid handling and utilization after SG, reinforcing the idea that caloric restriction promotes a shift toward reliance on such pathways [28].

Changes to aspartate, tyrosine, and glycine suggest the interplay between successful long-term weight loss and amino acid metabolism in the gut. Notably, while lipid-associated species comprised the majority of significant metabolites, aspartate emerged as the most statistically significant perturbed metabolite (*p* = 0.004). Guo et al. [29] identified L-aspartate as an endogenous regulator of energy expenditure and suppressor of adipogenesis and lipogenesis. In high-fat and high-cholesterol diet-induced obese C57BL/6 mice, circulating and adipose L-aspartate levels were inversely correlated with obesity severity, with oral L-aspartate supplementation (200 mg/kg) reducing weight gain and improving metabolic parameters [29]. Although we report the significance of aspartate without stereochemical assignment, its prominence in our fecal metabolomics data is consistent with existing evidence linking aspartate metabolism to obesity, suggesting a promising target for intervention in the management of obesity and successful weight loss outcomes after bariatric surgery.

Linear modeling comparing baseline and 12-month post-operative serum metabolomic profiles revealed ten significantly altered metabolites in super-responsive (T_3_) patients. This metabolic signature was predominantly driven by lipid-related species, particularly phosphatidylcholines and sphingomyelins, with additional contributions from two non-lipid metabolites, creatine and citric acid, which exhibited the strongest statistical association in serum (*p* = 0.0005). Although lipid alterations were initiated early, their levels continued to evolve. In contrast, a subset of metabolite and lipid biomarkers exhibited a delayed response, becoming evident only at the 12-month time point. These late-stage changes were enriched in pathways related to energy metabolism and phosphatidylcholines, suggesting a secondary metabolic adaptation likely driven by preceding physiological changes such as weight loss. Prior work examining metabolite changes during structured low-calorie diet interventions with a subsequent maintenance phase demonstrated that shifts in circulating citric acid and lipid species are associated with greater reductions in body weight and adiposity [30]. Specifically, increases in citric acid during the weight-loss phase were linked to decreased subsequent adiposity regain during the maintenance phase [30]. Investigators proposed that rising citric acid concentrations may reflect weight loss-related bone turnover, releasing citrate from bone into the circulation [30]. Notably, early weight-loss interventions are associated with increases in osteocalcin, consistent with a catabolic bone state, further supporting the plausibility of such a mechanism [31]. The link between serum citric acid and lipid species and weight loss success after SG observed in our study is consistent with diet-based evidence associating these metabolites with reductions in adiposity and risk of regain, suggesting these metabolic signatures are consistent across both diet-induced and surgical weight loss [30].

Although these general patterns provide a useful foundation, sex-stratified metabolomics analyses remain indispensable for fully elucidating the variability in metabolic responses following SG. Biological sex influences adipose distribution, hormonal regulation, substrate utilization, and inflammatory pathways that shape postoperative metabolic remodeling and weight loss outcomes [32]. For instance, sex hormones such as estrogen and testosterone modulate lipid metabolism, insulin sensitivity, and mitochondrial function, leading to distinct metabolic responses. Pooling sexes may therefore obscure meaningful metabolic differences and reduce the ability to detect clinically relevant signals. These findings support the importance of incorporating sex as a biological variable in metabolomics analyses. Identifying sex-specific metabolic signatures aligns with a precision medicine framework by improving the prediction of weight loss outcomes and informing individualized postoperative strategies [33]. Integrating sex-stratified analyses may therefore enhance both mechanistic insight and clinical translation in bariatric metabolomics research. Thus, we performed sex-stratified analysis to identify sex-specific patterns that may inform individualized risk stratification and targeted interventions.

Within the sex-stratified serum analyses among females in T_1_, three phosphatidylcholine species (PC.aa.C34.3, PC.aa. C32.1, and PC.aa.C36.5) were significantly decreased at 12 months relative to baseline, whereas acetone was significantly increased. In females within T2, 3-hydroxybutyric acid was significantly elevated at 12 months compared to baseline. No significant serum metabolites were identified in T_3_. Although these metabolites cannot be considered unique to females, they participate in pathways known to be influenced by sex-related physiology. In particular, phosphatidylcholine metabolism is affected by hormonal regulation of lipid synthesis and remodeling, while acetone and 3-hydroxybutyric acid may reflect greater routing of fatty acids toward hepatic oxidation and ketone-body production in females [32,34,35]. These sex-stratified metabolic patterns may also be influenced by sex-related differences in dietary intake, adherence, and physical activity, as prior work has shown that metabolic responses to such lifestyle interventions can differ between men and women [36].

Such analysis is further supported by the existing literature comparing metabolic signatures of adiposity indices by sex. One study found that in females, metabolic signatures consisted of lipoprotein particle concentrations, apolipoproteins, and fatty acids, while males were rich in cholesteryl esters and aromatic/branched-chain amino acids [37]. However, large population-based studies have not consistently demonstrated clear sexual dimorphism in metabolite–adiposity associations. This inconsistency may be attributable to heterogeneity in population characteristics, differences in lifestyle and environmental exposures, as well as methodological limitations that could obscure subtle, sex-specific metabolic effects [38,39]. Collectively, these findings highlight the need to more precisely delineate how sex modulates metabolic signatures associated with weight loss, thereby advancing a precision medicine framework for SG and enabling the development of tailored adjunct therapies to optimize weight loss.

Because individual metabolite associations can be difficult to interpret in isolation, we performed functional metabolite analysis to highlight pathway-level signals to provide a broader view of the biological processes associated with weight loss success. Among T_3_ patients, the metabolic pathways that harbor significantly altered metabolites as identified within fecal samples, in order of descending statistical significance, include lysine metabolism, glutamate metabolism, phosphatidylcholines, purine metabolism, TCA cycle, and monoamine metabolism.

Functional metabolite analysis identified lysine and glutamate metabolism as the most significant fecal pathway-level signals in high responders, suggesting weight loss may be accompanied by broader shifts in gut amino-acid handling rather than limited to a few individual metabolites. Prior work in obesity has reported higher circulating lysine while noting that lysine restriction may ameliorate obesity, potentially through suppression of adipogenesis and microbiome-related mechanisms [40,41]. Our finding of altered lysine metabolism in feces is therefore compatible with postoperative changes in the gut metabolome that may influence lysine availability and microbial lysine metabolism. Similarly, glutamate has been linked to adiposity and central fat accumulation in systemic studies [42]. Fecal glutamate-pathway signaling is underexplored and here may capture changes in microbial amino acid metabolism and nitrogen handling that accompany long-term weight loss post-SG.41.

Purine metabolism also emerged as a significant signal in fecal profiles. One explanation for this finding may reflect a reversal of the metabolic changes described in obesity. Interestingly, visceral fat accumulation is closely associated with elevated uric acid, the principal end product of purine catabolism. This is due, in part, to elevated xanthine oxidoreductase activity in adipose tissue, which augments the production and secretion of uric acid. In contrast, our findings suggest that sustained weight loss is associated with a shift in purine metabolism, as corroborated by the extensively documented decrement in serum uric acid that occurs after weight loss, even post-bariatric surgery [43,44]. Some explanations for alterations to this pathway include the enhancement of insulin sensitivity, which improves renal function and aids in the clearance of uric acid, as well as changes to the gut microbiota after surgery, which have been linked to intestinal uric acid clearance [45,46]. Another explanation includes the influence of sex-related differences in gut microbial composition and function, which can alter microbial metabolite production and contribute to variability in host metabolic response [47]. Other fecal metabolic pathway alterations, as aforementioned, include phosphatidylcholine metabolism, the TCA cycle, and monoamine metabolism, suggesting concurrent shifts in gut lipid composition and energy metabolism.

Functional metabolite analysis of serum profiles was dominated by lipid classes, with sphingomyelins (SMs), lysophosphatidylcholines (LPCs), and phosphatidylcholines (PCs) emerging as the most significant. The three major phospholipids found in humans each play important roles in the modulation of membrane-enzyme activity as well as lipid turnover and signaling [48]. Previous quantitative plasma lipid profiling reported SM, LPC, and PC as the most abundant lipid classes in both lean and obese individuals, with LPC levels noted to be lower in obesity and inversely correlated with BMI and inflammatory markers [49]. Our serum functional analysis supports phospholipid remodeling as a key biochemical signature associated with postoperative weight loss outcomes after SG.

The findings of this study are important for understanding the mechanisms by which SG improves metabolic characteristics in patients with obesity. By identifying potential metabolic biomarkers, such as aspartate, citrate, tyrosine, glycine, and various lipid species, this study provides a scientific basis for predicting surgical outcomes and developing novel therapeutic strategies. Moreover, as presented in our 3-month outcomes study, these metabolic biomarkers have great potential to develop machine learning models for clinical application, assisting in postoperative follow-up monitoring and the formulation of individualized treatment plans following SG [12]. Integrating metabolomic biomarkers into predictive models aligns with the goals of precision medicine to optimize treatment for each patient. It is worth noting that the feature selection algorithm delivered different metabolite panels at 3 and 12 months for prediction of weight loss, while machine learning algorithms found that both preoperative serum and fecal metabolites were highly predictive of weight loss outcomes, specifically for the highest weight loss group.

Moreover, both embedded and non-embedded machine learning approaches demonstrated comparable predictive performance, suggesting that the underlying metabolic signal associated with weight loss outcomes is robust and can be effectively captured by multiple modeling strategies. Specifically, the similar performance of regularization-based models such as GLMNET, which incorporate embedded feature selection, and algorithms such as SVM, which rely on external or implicit feature selection, indicates that model performance was not strongly dependent on the feature selection strategy alone. This convergence further implies that the discriminative metabolite patterns are sufficiently pronounced to be detected regardless of whether variable selection is performed intrinsically during model training or handled separately. Importantly, these machine learning analyses should be interpreted as retrospective classification and metabolic state characterization rather than prospective prediction models. In particular, models incorporating 12-month metabolomic data distinguish metabolic profiles associated with concurrent post-operative response groups, but do not independently predict 12-month weight loss outcomes before clinically apparent. The greater clinical value of this approach will depend on future studies designed to determine whether preoperative or early postoperative metabolomic features can predict later weight-loss trajectories. Taken together, these findings highlight the stability of the identified metabolic signatures and support their potential utility in predictive modeling, while also suggesting that both model classes may be suitable for developing clinically relevant tools for the prediction of weight loss following SG.

Notably, across both time points, classification models derived from serum metabolomics consistently outperformed those based on fecal metabolomics data. This disparity likely reflects fundamental differences in data stability and biological variability between the two matrices. Longitudinal metabolomics studies are inherently susceptible to batch effects; however, fecal metabolomics are further confounded by substantial intra- and inter-individual variability driven by factors such as dietary fluctuations, bowel transit time, microbiome dynamics, and sample heterogeneity. In contrast, serum metabolite profiles are generally more stable, tightly regulated, and reproducible, thereby providing a more consistent representation of systemic metabolic status. Consequently, serum metabolomics is better suited for robust predictive modeling and biomarker discovery. In light of these considerations, serum data was prioritized for the development of more complex models, whereas fecal data was leveraged to construct simpler models, such as those based on logistic regression, to account for their higher variability and lower signal consistency.

Our novel metabolomics study presented here is not free of limitations. First, longitudinal studies have inherent limitations, one such constraint being that fecal and serum samples must be collected at widely spaced time intervals, which can result in unavoidable variations in the data collection and sample preparation [50]. Ideally, all samples would be processed and analyzed in a single batch to maintain consistency of the extraction protocol, but the year-long collection schedule makes this impossible. Another limitation is the small sample size of the study and the predominantly female and Caucasian cohort, including only five male participants, which limits the ability to generalize findings across different groups and precludes robust conclusions regarding SG-associated metabolomic changes in male patients with obesity. As mentioned in our 3-month analysis, future studies should evaluate larger, more sex-balanced cohorts, or all female and all male cohorts, to minimize the influence of gender on weight loss outcomes and more clearly define signatures that could inform a precision-based approach to SG [12]. Additionally, the comparison between the highest and lowest weight loss tertiles may be influenced by anthropometric differences, particularly baseline BMI, which differed between T3 and T1. Although statistical adjustment was used to mitigate unwanted variation, baseline BMI and subsequent weight-loss response are intrinsically related. Therefore, the observed metabolomic profiles may reflect a combination of baseline metabolic state and biologic response to SG, rather than surgical response alone. Similarly, the machine learning analyses should be interpreted as exploratory given the modest overall sample size. Although cross-validation was used to estimate model performance, the limited cohort size increases the risk of overfitting, and the reported AUC values may represent optimistic estimates of discriminatory performance. Larger cohorts will be needed to validate these findings before such models can be considered clinically actionable. Finally, we could not impose or monitor the diet of participants or their medication history for twelve months. Variations in dietary intake or medication have been known to modulate the pathways highlighted in our analysis and thus may have introduced additional variability into the metabolomic profiles of the fecal and serum samples of participants.

## 5. Conclusions

Despite the acknowledged limitations, this study represents the first comprehensive multi-platform, multi-compartment longitudinal metabolomics investigation, providing a robust scientific framework for predicting surgical outcomes following SG and for guiding the development of novel therapeutic strategies. The identified metabolic biomarkers hold significant clinical potential, particularly for postoperative monitoring and the design of personalized treatment approaches.

Future studies should aim to include larger cohorts, extend follow-up durations, and integrate additional omics technologies to further validate and expand upon these findings. Such efforts will facilitate a more comprehensive understanding of the metabolic effects of SG, particularly in patients with comorbid conditions such as obesity and type 2 diabetes mellitus. Overall, this work contributes valuable insights and establishes a foundation for the translation of metabolomics-based biomarkers into clinical practice.

## Figures and Tables

**Table 1:** Baseline demographics, anthropometric measures, and comorbidities, with comparison of the highest %TWL tertile (T_3_) with the lowest %TWL tertile (T_1_) [12].

	Cohort (*n* = 45)	T3 (*n* = 15)	T1 (*n* = 15)	*p*-Value (T3 vs. T1)
Age (years)	48.2 ± 11.5	47.3 ± 12.1	51.0 ± 9.4	0.35
Weight (kg)	125.7 ± 20.6	123.8 ± 25.1	130.6 ± 18.7	0.38
BMI (kg/m^2^)	45.3 ± 7.3	42.0 ± 5.4	48.8 ± 8.7	0.02
Gender (female)	89%	73%	100%	0.10
Race				
- White	60%	73%	60%	0.43
- Black	36%	20%	40%	
- Two races	2%	7%	0%	
- Native American	2%	0%	0%	
Diabetes	29%	13%	40%	0.22
Dyslipidemia	56%	53%	47%	0.72
Hypertension	64%	53%	80%	0.12

Note: %TWL = percent total weight loss.

## Data Availability

The metabolomics data represented in this exploratory study will be available upon request. Data is not publicly available due to privacy.

## Supplementary Materials

The following supporting information can be downloaded at https://www.mdpi.com/article/10.3390/metabo16070497/s1: Supplementary Methods information; **Table S1:** Log-scaled mean concentration ratio of fecal metabolites, along with the results of linear models comparing metabolic profile differences for the pairwise comparison of BL versus 12M_T3; **Table S2:** Log-scaled mean concentration ratio of serum metabolites, along with the results of linear models comparing metabolic profile differences for the pairwise comparison of BL versus 12M_T3; **Table S3:** Log-scaled mean concentration ratio of fecal metabolites, along with results from sex-stratified linear models comparing metabolic profile differences for the pairwise comparison of BL versus 12M_T3; **Table S4:** Log-scaled mean concentration ratio of serum metabolites, along with results from sex-stratified linear models comparing metabolic profile differences for the pairwise comparison of BL versus 12M_T3; **Figure S1:** Quality-control assessment of longitudinally collected fecal metabolomics data using principal component analysis (PCA) to determine the existence of systematic variation, outliers, and potential batch effects (a) before and (b) after data preprocessing; **Figure S2:** Quality-control assessment of longitudinally collected serum metabolomics data by principal component analysis (PCA) to identify systematic variation, outliers, and potential batch effects (a) before and (b) after data preprocessing; **Figure S3:** Histogram graphs displaying the overall signal intensity distribution across fecal metabolomics datasets before (A) and after (B) preprocessing, with aggregated conditions (a) and corresponding group-specific density plots of the normalized signal profiles (b); **Figure S4:** Histogram graphs displaying the overall signal intensity distribution across serum metabolomics datasets before (A) and after (B) preprocessing, with aggregated conditions (a) and corresponding group-specific density plots of the normalized signal profiles (b); Histograms display; **Figure S5:** Hierarchical clustering and heatmap of Pearson correlations between all covariates incorporated into linear models based on fecal metabolomics data, so that covariates with higher correlations will be dropped from the reduced model; **Figure S6:** Hierarchical clustering and heatmap of Pearson correlations between all covariates incorporated into linear models based on serum metabolomics data, so that covariates with higher correlations will be dropped from the reduced model; **Figure S7:** Volcano plots showing the results of sex-stratified analyses (i–ii) for both fecal (A) and serum (B) metabolomics data; **Figure S8:** Correlations among the 30 most significantly perturbed metabolites at each time point (BL, 3M, and 12M) for fecal (A) and serum (B) metabolomics data obtained from patients who underwent SG.
