# Supplementary material for: Longitudinal Assessment of Twelve-Month Weight Loss Outcomes Post-Sleeve Gastrectomy: The Role of Serum and Fecal Metabolomic Biomarkers
Source: Metabolites. 2026 Jul 15;16(7):497. doi: 10.3390/metabo16070497 (PMC13414149; doi:10.3390/metabo16070497)
Supplement: Supplementary file 1 [file metabolites-16-00497-s001.zip › metabolites-4344167-supplementary.pdf]

Longitudinal Assessment of Twelve-Month Weight Loss Outcomes Post-Sleeve Gastrectomy: The Role of Serum and Fecal Metabolomic Biomarkers

Maya Nassif [1,*], Wendy M. Miller [1,2,*], Kathryn M. Ziegler [1,2], Nadia Ashrafi [3], Romana Mimi Ashrafi [3], Abdullah Khalid [3], Sumeyya Akyol [3], Jay Idler [4,5], Milda Milčiūtė [6], Vilija Lomeikaitė [6], Austėja Jankevičiūtė [6], Matthew D. Sims [1,2,3], Michael E. Maddens [1,2], Ali Yilmaz [1,2,3] and Stewart F. Graham [1,2,3]

[1] Oakland University William Beaumont School of Medicine, 586 Pioneer Dr, Rochester, MI 48309, USA; mat ali.yilmaz@corewellhealth.org (A.Y.); stewart.graham@corewellhealth.org (S.F.G.)
[2] Corewell Health William Beaumont University Hospital, 3601 W. 13 Mile Road, Royal Oak, MI 48073, USA
[3] Corewell Health Research Institute, 3811 W 13 Mile Rd, Royal Oak, MI 48073, USA
[4] West Penn Hospital, Allegheny Health Network, 4815 Liberty Ave, Suite GR50, Pittsburgh, PA 15224, USA
[5] Drexel University College of Medicine, 2900 W Queen Ln, Philadelphia, PA 19129, USA
[6] VUGENE, LT-10312 Vilnius, Lithuania; austeja@vugene.com (A.J.)
* Correspondence: mnassif@oakland.edu (M.N.); wendy.miller@corewellhealth.org (W.M.M.); Tel.: +1-248-655-5934 (W.M.M.)

Table S1. Log scaled mean concentration ratio of the fecal metabolites along with the results of the linear models comparing metabolic profile differences for pair-wise comparison for BL vs. 12M_T_3.

Metabolites	logFC	t	P.Value
Ac.Orn	0.400259	0.854564	0.394653
Acetic.acid	-0.2735	-0.81959	0.414223
Acetone	-0.26811	-0.79049	0.430948
Ala	0.100653	0.175935	0.860669
alpha.AAA	0.996341	1.741351	0.084423
Asp	0.176372	0.438393	0.661962
Aspartate	1.037889	2.91251	0.004344
Betaine	-0.38253	-0.89978	0.370208
Butyrate	-0.52565	-1.1384	0.257429
C0	0.570768	2.252885	0.026254
C10.1	-0.00283	-0.01442	0.988518
C10.2	-0.24877	-0.98091	0.328791
C12	-0.01645	-0.09076	0.92785
C12.1	0.054921	0.279632	0.780286
C12.DC	-0.18271	-0.81743	0.415456
C14	-0.24604	-1.05507	0.293708
C14.1	-0.01525	-0.05422	0.956855
C14.1.OH	0.110037	0.373202	0.709718
C14.2	0.054869	0.245786	0.806307
C14.2.OH	-0.27408	-1.18088	0.240203
C16	-0.2131	-0.59258	0.554679
C16.1	-0.17621	-0.58094	0.562475
C16.1.OH	-0.00223	-0.00441	0.996489
C16.2	-0.15276	-0.58414	0.560327
C16.2.OH	-0.36993	-1.57283	0.118635
C16.OH	-0.09807	-0.16673	0.867886
C18	-0.35298	-0.92073	0.359209
C18.1	-0.4271	-1.10216	0.272802
C18.1.OH	-0.52261	-1.60944	0.110391
C18.2	-0.18268	-0.4736	0.636726
C2	-0.16823	-0.66789	0.505605
C3.DC..C4.OH.	0.119063	0.33412	0.738926
C3.OH	-0.02214	-0.1132	0.910077
C4.1	-0.03152	-0.17887	0.858369
C5	-0.20587	-0.90157	0.369257
C5.DC..C6.OH.	-0.03803	-0.16395	0.87007
C5.M.DC	-0.20542	-0.8925	0.374077
C5.OH..C3.DC.M.	-0.2438	-0.87472	0.383637
C7.DC	-0.05755	-0.15106	0.880206
C9	-0.08351	-0.30804	0.758633
Choline	0.087864	0.250887	0.80237
Cit	-0.17278	-0.58073	0.562615
Creatine	-0.50162	-1.31389	0.191621

Creatinine	-1.03957	-1.37465	0.172037
D.Galactose	0.355788	1.069898	0.287011
D.Glucose	-1.09626	-1.9533	0.053329
Dimethylamine	0.593604	1.38923	0.167574
Ethanol	-0.16868	-0.44185	0.65947
Formate	0.286809	1.188477	0.23721
Fumaric.acid	0.275948	0.796632	0.427384
Gln	0.495057	1.29368	0.19849
Glu	0.734688	1.371391	0.173049
Gly	-0.23256	-0.71485	0.476219
Glycerol	-0.13595	-0.2433	0.808229
Glycine	0.674018	2.177337	0.031596
His	-0.48773	-1.41334	0.160384
Hypoxanthine	0.024781	0.068039	0.945878
Isobutyric.acid	-0.58196	-0.98849	0.325086
Isoleucine	0.221871	0.556778	0.578812
Isopropyl.alcohol	-0.05864	-0.19073	0.84909
Isovaleric.acid	0.090859	0.180228	0.857306
L.Fucose	-0.27957	-0.83751	0.404127
L.Glutamic.acid	0.792297	1.612603	0.109701
L.Lactic.acid	-0.19735	-0.45039	0.653319
L.Leucine	-0.02151	-0.05076	0.959606
L.Lysine	0.613996	1.458183	0.147644
L.Phenylalanine	0.093958	0.318798	0.750485
lysoPC.a.C14.0	0.128895	0.536064	0.592999
lysoPC.a.C16.0	-0.1449	-0.38559	0.700544
lysoPC.a.C16.1	-0.07103	-0.33237	0.740244
lysoPC.a.C17.0	-0.01554	-0.09158	0.927197
lysoPC.a.C18.0	-0.39384	-1.44672	0.150824
lysoPC.a.C18.1	-0.25209	-0.51924	0.60464
lysoPC.a.C18.2	-0.3597	-0.68619	0.494038
lysoPC.a.C20.4	-0.03906	-0.17105	0.8645
lysoPC.a.C24.0	-0.07928	-0.31655	0.752189
lysoPC.a.C26.0	-0.12695	-0.78154	0.436166
lysoPC.a.C26.1	-0.09346	-0.53794	0.591704
lysoPC.a.C28.0	-0.03828	-0.28038	0.779713
lysoPC.a.C28.1	-0.13239	-0.79274	0.429638
Met.SO	-0.01018	-0.03256	0.974086
Methanol	-0.58977	-0.94185	0.348338
Methionine	0.31347	1.012428	0.313559
Methylamine	0.266457	0.900535	0.369806
Orn	-0.22559	-0.55398	0.580719
PC.aa.C24.0	-0.2435	-1.60977	0.11032
PC.aa.C28.1	0.19153	1.006376	0.316447
PC.aa.C30.0	-0.02931	-0.12581	0.900114
PC.aa.C32.0	-0.08046	-0.40944	0.683013

PC.aa.C32.1	-0.42028	-1.23068	0.221072
PC.aa.C32.2	-0.36039	-1.67567	0.096647
PC.aa.C32.3	-0.09817	-0.52954	0.597499
PC.aa.C34.1	0.027147	0.077853	0.938087
PC.aa.C34.2	-0.18845	-0.59636	0.552162
PC.aa.C34.3	-0.03361	-0.13661	0.891593
PC.aa.C34.4	0.309729	1.71234	0.089657
PC.aa.C36.0	0.263168	1.555779	0.122638
PC.aa.C36.1	0.286643	0.810321	0.419508
PC.aa.C36.2	-0.06954	-0.23502	0.814628
PC.aa.C36.3	0.049819	0.174955	0.861437
PC.aa.C36.4	0.033837	0.100347	0.920252
PC.aa.C36.5	-0.08202	-0.35337	0.724492
PC.aa.C36.6	-0.15717	-0.7942	0.428795
PC.aa.C38.0	0.394052	2.059656	0.041796
PC.aa.C38.3	0.095986	0.420863	0.674678
PC.aa.C38.4	0.51694	1.975242	0.05075
PC.aa.C38.5	0.109119	0.334424	0.738697
PC.aa.C38.6	-0.35249	-1.48676	0.139943
PC.aa.C40.1	0.101389	0.711362	0.478368
PC.aa.C40.2	-0.18514	-0.64859	0.517955
PC.aa.C40.3	0.244469	1.00039	0.319321
PC.aa.C40.4	0.234575	1.046912	0.297439
PC.aa.C40.5	0.442817	2.129879	0.035415
PC.aa.C40.6	0.048336	0.24737	0.805083
PC.aa.C42.0	-0.14404	-1.05562	0.293461
PC.aa.C42.1	0.236437	1.574985	0.118137
PC.aa.C42.2	-0.01242	-0.08262	0.934304
PC.aa.C42.4	0.130299	0.645751	0.519787
PC.aa.C42.5	0.097788	0.446443	0.656156
PC.aa.C42.6	0.140334	0.503767	0.615434
PC.ae.C30.0	-0.15401	-1.00083	0.319111
PC.ae.C30.1	-0.35776	-1.57431	0.118294
PC.ae.C30.2	0.161349	0.956178	0.341083
PC.ae.C32.1	-0.06377	-0.25361	0.800274
PC.ae.C32.2	0.15791	0.850671	0.396803
PC.ae.C34.0	0.209006	0.995651	0.321609
PC.ae.C34.1	-0.09186	-0.32897	0.742802
PC.ae.C34.2	0.198543	0.673025	0.502345
PC.ae.C34.3	0.343781	1.525967	0.129893
PC.ae.C36.0	0.250343	1.271979	0.206068
PC.ae.C36.1	-0.03035	-0.18768	0.851477
PC.ae.C36.2	-0.1	-0.58293	0.561136
PC.ae.C36.3	-0.01541	-0.09284	0.926201
PC.ae.C36.4	0.572782	2.589501	0.010914
PC.ae.C36.5	0.164193	0.88339	0.378956

PC.ae.C38.0	0.018785	0.131449	0.895661
PC.ae.C38.1	0.053647	0.300396	0.764443
PC.ae.C38.2	-0.22261	-0.82	0.41399
PC.ae.C38.3	0.114858	0.718653	0.473881
PC.ae.C38.4	0.340111	1.680217	0.095758
PC.ae.C38.5	0.066716	0.32821	0.743378
PC.ae.C38.6	0.052486	0.203437	0.83917
PC.ae.C40.1	-0.24636	-1.25259	0.213017
PC.ae.C40.2	0.492202	2.316367	0.022394
PC.ae.C40.3	0.243827	1.177222	0.241652
PC.ae.C40.4	0.134165	0.786901	0.433035
PC.ae.C40.5	0.294902	1.299118	0.196625
PC.ae.C40.6	-0.14179	-0.67448	0.501425
PC.ae.C42.0	0.001899	0.013256	0.989448
PC.ae.C42.1	-0.26697	-1.86443	0.064932
PC.ae.C42.2	0.270754	1.395293	0.165743
PC.ae.C42.3	-0.16532	-0.91026	0.36468
PC.ae.C42.5	-0.04878	-0.32766	0.743795
PC.ae.C44.3	0.087705	0.522066	0.602676
PC.ae.C44.4	-0.2932	-2.0023	0.047717
PC.ae.C44.5	-0.15319	-0.91056	0.364522
PC.ae.C44.6	-0.05288	-0.39193	0.695868
Phe	-0.50661	-1.28197	0.202552
Phenylacetate	-0.00273	-0.01031	0.991789
Pro	0.056522	0.1421	0.887261
Putrescine	-1.09377	-1.10198	0.272878
Pyruvic.acid	0.252754	0.676827	0.499939
Sarcosine	-0.31616	-0.85997	0.391677
SDMA	0.667387	1.301211	0.19591
Ser	0.099537	0.346333	0.729755
Serotonin	0.553396	1.384663	0.168963
SM..OH..C14.1	0.1514	0.437739	0.662435
SM..OH..C16.1	-0.01216	-0.02979	0.976289
SM..OH..C22.1	-0.19242	-0.50664	0.613423
SM..OH..C22.2	0.30636	0.812006	0.418545
SM..OH..C24.1	0.549815	1.671283	0.097513
SM.C16.0	0.419283	1.007495	0.315912
SM.C16.1	-0.01056	-0.0552	0.956082
SM.C18.0	0.344703	0.599185	0.550283
SM.C18.1	0.206453	0.513915	0.608344
SM.C20.2	-0.1074	-0.55482	0.580147
SM.C24.0	0.120487	0.462227	0.644833
SM.C26.0	0.192661	0.765505	0.445613
Spermidine	-0.64772	-0.9842	0.327179
Succinate	0.656397	1.107191	0.270631
t4.OH.Pro	0.133807	0.258465	0.796532

Taurine	-0.56894	-0.84119	0.402071
Thr	-0.02307	-0.11275	0.910438
Trimethylamine	0.243502	0.616219	0.539025
Trp	-0.18575	-0.67134	0.503416
Tyr	-0.74814	-2.41891	0.017211
Tyrosine	0.085568	0.328502	0.743158
Uracil	0.263061	0.830405	0.408112
Valine	0.639729	1.284223	0.201767
3-Hydroxyisovaleric.acid	0.061613	0.133413	0.894111
Xanthine	-0.30054	-0.82871	0.409067

Table S2. Log scaled mean concentration ratio of the serum metabolites along with the results of the linear models comparing metabolic profile differences for pair-wise comparison for BL vs. $12M\_T_3$.

ID	logFC	t	Contrast
Acetic.acid	0.258297	0.965598	M12-3vsBL
Acetoacetate	0.552132	1.423484	M12-3vsBL
Acetone	0.488814	1.674781	M12-3vsBL
ADMA	0.012768	0.04201	M12-3vsBL
alpha.AAA	-0.59738	-1.83814	M12-3vsBL
Asp	-0.14155	-0.97781	M12-3vsBL
Betaine	-0.00374	-0.01846	M12-3vsBL
C0	-0.16019	-1.22811	M12-3vsBL
C10	-0.42699	-1.50531	M12-3vsBL
C14.1	-0.05676	-0.33209	M12-3vsBL
C16	-0.05723	-0.52187	M12-3vsBL
C18	0.15804	0.217777	M12-3vsBL
C18.1	0.143749	1.047692	M12-3vsBL
C18.2	-0.00287	-0.01919	M12-3vsBL
C2	-0.35142	-1.87092	M12-3vsBL
C3	-0.09257	-0.47692	M12-3vsBL
C3.DC..C4.OH.	0.169784	0.837921	M12-3vsBL
C4	-0.0793	-0.45473	M12-3vsBL
C5	0.026851	0.128542	M12-3vsBL
Carnitine	-0.19524	-0.91371	M12-3vsBL
Choline	-0.11051	-0.40706	M12-3vsBL
Cit	0.111765	0.746818	M12-3vsBL
Citric.acid	0.563295	3.595332	M12-3vsBL
Creatine	-0.55379	-2.17113	M12-3vsBL
Creatinine	-0.19908	-1.07551	M12-3vsBL
D.Glucose	0.00014	0.000754	M12-3vsBL
Dimethyl.sulfone	0.033696	0.11297	M12-3vsBL
Ethanol	-0.03064	-0.1238	M12-3vsBL
Formate	-0.06563	-0.31669	M12-3vsBL
Glu	-0.20944	-1.01467	M12-3vsBL
Gly	-0.0087	-0.05262	M12-3vsBL
Glycerol	0.036529	0.210176	M12-3vsBL
H1	-0.06298	-0.84634	M12-3vsBL
Hypoxanthine	0.020035	0.064502	M12-3vsBL
Isobutyric.acid	-0.10879	-0.45366	M12-3vsBL
Isoleucine	-0.12211	-0.57694	M12-3vsBL
Isopropyl.alcohol	-0.01857	-0.0648	M12-3vsBL
Kynurenine	0.143827	0.938776	M12-3vsBL
L.Alanine	-0.0461	-0.20562	M12-3vsBL
L.Arginine	0.46983	1.750519	M12-3vsBL
L.Histidine	-0.1918	-0.62578	M12-3vsBL
L.Lactic.acid	-0.00896	-0.05039	M12-3vsBL
Leu	-0.12877	-1.04333	M12-3vsBL

Lys	0.015873	0.149345	M12-3vsBL
lysoPC.a.C16.0	-0.10287	-0.82109	M12-3vsBL
lysoPC.a.C16.1	-0.03188	-0.20976	M12-3vsBL
lysoPC.a.C17.0	0.071928	0.548916	M12-3vsBL
lysoPC.a.C18.0	-0.11946	-0.99742	M12-3vsBL
lysoPC.a.C18.1	0.08926	0.623764	M12-3vsBL
lysoPC.a.C18.2	0.072303	0.44411	M12-3vsBL
lysoPC.a.C20.3	-0.1327	-0.83188	M12-3vsBL
lysoPC.a.C20.4	0.016887	0.111828	M12-3vsBL
lysoPC.a.C28.1	-0.21734	-1.31697	M12-3vsBL
Malonate	0.058871	0.201498	M12-3vsBL
Met	0.100566	0.622426	M12-3vsBL
Met.SO	0.126066	0.242087	M12-3vsBL
Orn	0.036823	0.23046	M12-3vsBL
PC.aa.C28.1	-0.01712	-0.1867	M12-3vsBL
PC.aa.C30.0	0.215694	1.736592	M12-3vsBL
PC.aa.C32.0	0.241972	2.749481	M12-3vsBL
PC.aa.C32.1	0.0105	0.058693	M12-3vsBL
PC.aa.C32.2	-0.28711	-1.6643	M12-3vsBL
PC.aa.C32.3	-0.02289	-0.20414	M12-3vsBL
PC.aa.C34.1	0.015819	0.118987	M12-3vsBL
PC.aa.C34.2	-0.09788	-1.00245	M12-3vsBL
PC.aa.C34.3	-0.16331	-1.38418	M12-3vsBL
PC.aa.C34.4	-0.45813	-3.17395	M12-3vsBL
PC.aa.C36.0	-0.00348	-0.0137	M12-3vsBL
PC.aa.C36.1	-0.11509	-0.85535	M12-3vsBL
PC.aa.C36.2	-0.1893	-1.40671	M12-3vsBL
PC.aa.C36.3	-0.18628	-1.50922	M12-3vsBL
PC.aa.C36.4	-0.08338	-0.7141	M12-3vsBL
PC.aa.C36.5	-0.27286	-1.81285	M12-3vsBL
PC.aa.C36.6	-0.55597	-2.83532	M12-3vsBL
PC.aa.C38.0	0.018015	0.16817	M12-3vsBL
PC.aa.C38.3	9.82E-05	0.000677	M12-3vsBL
PC.aa.C38.4	-0.16077	-1.56056	M12-3vsBL
PC.aa.C38.5	-0.11078	-0.97265	M12-3vsBL
PC.aa.C38.6	-0.16103	-1.27081	M12-3vsBL
PC.aa.C40.1	0.145863	1.575767	M12-3vsBL
PC.aa.C40.4	0.060226	0.419766	M12-3vsBL
PC.aa.C40.5	0.02749	0.186198	M12-3vsBL
PC.aa.C40.6	-0.23439	-1.68309	M12-3vsBL
PC.aa.C42.0	0.063457	0.514468	M12-3vsBL
PC.aa.C42.1	-0.00275	-0.02082	M12-3vsBL
PC.aa.C42.5	0.149759	1.255335	M12-3vsBL
PC.aa.C42.6	0.004842	0.042515	M12-3vsBL
PC.ae.C30.0	0.007075	0.05248	M12-3vsBL
PC.ae.C30.1	0.058723	0.311742	M12-3vsBL

PC.ae.C32.1	0.321441	3.3308	M12-3vsBL
PC.ae.C32.2	0.180679	1.991828	M12-3vsBL
PC.ae.C34.0	0.122277	1.297482	M12-3vsBL
PC.ae.C34.1	0.173516	2.164244	M12-3vsBL
PC.ae.C34.2	0.113392	1.241861	M12-3vsBL
PC.ae.C34.3	0.248855	2.427522	M12-3vsBL
PC.ae.C36.0	0.091371	1.0023	M12-3vsBL
PC.ae.C36.1	-0.02435	-0.30989	M12-3vsBL
PC.ae.C36.2	0.065127	0.690372	M12-3vsBL
PC.ae.C36.3	0.036876	0.407702	M12-3vsBL
PC.ae.C36.4	-0.08081	-0.87159	M12-3vsBL
PC.ae.C36.5	0.06507	0.574687	M12-3vsBL
PC.ae.C38.0	0.003051	0.021687	M12-3vsBL
PC.ae.C38.1	-0.44721	-1.55393	M12-3vsBL
PC.ae.C38.2	0.061674	0.398763	M12-3vsBL
PC.ae.C38.3	0.022566	0.160493	M12-3vsBL
PC.ae.C38.4	-0.09353	-0.95366	M12-3vsBL
PC.ae.C38.5	-0.12325	-1.14109	M12-3vsBL
PC.ae.C38.6	-0.12685	-1.03061	M12-3vsBL
PC.ae.C40.1	-0.00267	-0.02603	M12-3vsBL
PC.ae.C40.2	0.016171	0.074247	M12-3vsBL
PC.ae.C40.3	0.091568	0.449785	M12-3vsBL
PC.ae.C40.4	0.126693	1.052615	M12-3vsBL
PC.ae.C40.5	0.079458	0.584011	M12-3vsBL
PC.ae.C40.6	-0.10411	-0.8306	M12-3vsBL
PC.ae.C42.1	2.77E-05	0.000211	M12-3vsBL
PC.ae.C42.2	0.052291	0.386458	M12-3vsBL
PC.ae.C42.3	-0.0669	-0.60849	M12-3vsBL
PC.ae.C42.4	0.211284	1.488854	M12-3vsBL
PC.ae.C42.5	0.115353	0.977065	M12-3vsBL
PC.ae.C44.3	0.056814	0.407585	M12-3vsBL
PC.ae.C44.4	0.099576	0.778469	M12-3vsBL
PC.ae.C44.5	0.109421	0.811395	M12-3vsBL
PC.ae.C44.6	0.094461	0.839718	M12-3vsBL
Pro	-0.05273	-0.41654	M12-3vsBL
Propylene.glycol	0.454426	1.169321	M12-3vsBL
Putrescine	-0.13306	-0.73037	M12-3vsBL
Pyruvic.acid	-0.3312	-1.34542	M12-3vsBL
Sarcosine	-0.03719	-0.17263	M12-3vsBL
SDMA	-0.00402	-0.0387	M12-3vsBL
Serotonin	-0.08813	-0.18337	M12-3vsBL
SM..OH..C14.1	0.088849	0.976494	M12-3vsBL
SM..OH..C16.1	0.100786	0.933373	M12-3vsBL
SM..OH..C22.1	-0.33664	-3.59449	M12-3vsBL
SM..OH..C24.1	-0.16073	-1.60381	M12-3vsBL
SM.C18.0	0.028275	0.2557	M12-3vsBL

SM.C18.1	0.109852	0.985995	M12-3vsBL
SM.C20.2	0.052195	0.35657	M12-3vsBL
SM.C24.1	0.169377	1.491239	M12-3vsBL
SM.C26.0	-0.05678	-0.40771	M12-3vsBL
SM.C26.1	0.231337	1.489675	M12-3vsBL
Succinate	0.106928	0.744386	M12-3vsBL
t4.OH.Pro	0.207874	0.820278	M12-3vsBL
Taurine	-0.08847	-0.57701	M12-3vsBL
Thr	0.174354	1.52997	M12-3vsBL
Trp	0.025345	0.163625	M12-3vsBL
Tyr	0.002185	0.013841	M12-3vsBL
Urea	0.047556	0.16729	M12-3vsBL
Val	-0.19636	-1.49047	M12-3vsBL
X1.Methylhistidine	-0.16199	-1.0373	M12-3vsBL
2-Hydroxybutyric.acid	0.207427	1.057081	M12-3vsBL
3-Hydroxybutyric.acid	0.708479	1.866956	M12-3vsBL

Table S3. Log-scaled mean concentration ratio of fecal metabolites, along with results from sex-stratified linear models comparing metabolic profile differences for the pairwise comparison of BL vs. 12M_T3.

Metabolites	logFC	t	P.Value	Sex
Ac.Orn	0.534569	0.982951	0.328071	female
Acetic.acid	-0.280294	-0.71851	0.474166	female
Acetone	-0.494512	-1.270999	0.206761	female
Ala	0.546298	0.833356	0.406686	female
alpha.AAA	1.129604	1.726214	0.087485	female
Asp	0.280534	0.59504	0.553199	female
Aspartate	0.825299	1.933025	0.056142	female
Betaine	-0.28737	-0.568219	0.571196	female
Butyrate	-0.593798	-1.103832	0.27239	female
C0	0.503297	1.573319	0.118893	female
C10.1	-0.13822	-0.643572	0.521368	female
C10.2	-0.177438	-0.650948	0.516616	female
C12	-0.081639	-0.391656	0.69617	female
C12.1	0.021019	0.095579	0.924051	female
C12.DC	-0.058825	-0.2158	0.829595	female
C14	-0.200261	-0.74157	0.460135	female
C14.1	-0.108597	-0.340121	0.734499	female
C14.1.OH	-0.009601	-0.024826	0.980244	female
C14.2	-0.099098	-0.371308	0.711216	female
C14.2.OH	-0.324723	-1.191647	0.2363	female
C16	-0.275409	-0.585295	0.559705	female
C16.1	-0.232884	-0.66395	0.508293	female
C16.1.OH	0.015172	0.0257	0.979549	female
C16.2	-0.166404	-0.524811	0.600909	female
C16.2.OH	-0.554657	-1.954579	0.053505	female
C16.OH	-0.051234	-0.073521	0.941543	female
C18	-0.452683	-1.021815	0.309404	female
C18.1	-0.376967	-0.789467	0.43176	female
C18.1.OH	-0.536299	-1.314763	0.191682	female
C18.2	-0.244265	-0.537964	0.591831	female
C2	-0.229288	-0.740045	0.461056	female
C3.DC..C4.OH.	0.091855	0.236099	0.813853	female
C3.OH	-0.084418	-0.395032	0.693684	female
C4.1	-0.018023	-0.093475	0.925718	female
C5	-0.27764	-1.023388	0.308664	female
C5.DC..C6.OH.	-0.109789	-0.428604	0.669159	female
C5.M.DC	-0.177702	-0.682405	0.496605	female
C5.OH..C3.DC.M.	-0.378332	-1.139244	0.2574	female
C7.DC	-0.10019	-0.232378	0.816733	female
C9	-0.18679	-0.572879	0.568049	female

**Metabolites**	**logFC**	**t**	**P.Value**	**Sex**
Choline	-0.024342	-0.060952	0.951522	female
Cit	-0.266633	-0.781131	0.436623	female
Creatine	-0.471063	-1.069223	0.287616	female
Creatinine	-0.913421	-1.034239	0.30359	female
D.Galactose	0.328872	0.842703	0.401463	female
D.Glucose	-1.650415	-2.575296	0.011522	female
Dimethylamine	0.700501	1.417323	0.159585	female
Ethanol	-0.255207	-0.572373	0.56839	female
Formate	0.41921	1.484509	0.140909	female
Fumaric.acid	0.184152	0.489005	0.625939	female
Gln	0.486827	1.081115	0.28232	female
Glu	1.219973	1.943552	0.054841	female
Gly	-0.218535	-0.603193	0.547785	female
Glycerol	0.505757	0.837084	0.404598	female
Glycine	0.766175	2.149522	0.034074	female
His	-0.506566	-1.296485	0.197877	female
Hypoxanthine	0.019797	0.046295	0.96317	female
Isobutyric.acid	-0.876196	-1.33914	0.183647	female
Isoleucine	0.073337	0.157299	0.875335	female
Isopropyl.alcohol	-0.152449	-0.42676	0.670497	female
Isovaleric.acid	-0.225398	-0.397576	0.691814	female
L.Fucose	-0.095071	-0.243974	0.807765	female
L.Glutamic.acid	0.816276	1.407072	0.162594	female
L.Lactic.acid	-0.225747	-0.4309	0.667495	female
L.Leucine	-0.097444	-0.199085	0.842612	female
L.Lysine	0.813493	1.680158	0.096137	female
L.Phenylalanine	0.000179	0.000538	0.999572	female
lysoPC.a.C14.0	0.249778	0.962584	0.338144	female
lysoPC.a.C16.0	-0.073319	-0.172811	0.863159	female
lysoPC.a.C16.1	-0.12837	-0.520014	0.604235	female
lysoPC.a.C17.0	-0.009023	-0.04863	0.961314	female
lysoPC.a.C18.0	-0.38135	-1.332801	0.185712	female
lysoPC.a.C18.1	-0.167323	-0.285333	0.775996	female
lysoPC.a.C18.2	-0.064492	-0.106717	0.915234	female
lysoPC.a.C20.4	0.097119	0.36852	0.713286	female
lysoPC.a.C24.0	-0.214536	-0.829801	0.408684	female
lysoPC.a.C26.0	-0.095624	-0.562058	0.57537	female
lysoPC.a.C26.1	-0.064385	-0.327405	0.744066	female
lysoPC.a.C28.0	-0.042995	-0.297964	0.766366	female
lysoPC.a.C28.1	-0.168655	-0.890618	0.375333	female
Met.SO	0.050461	0.135448	0.892538	female
Methanol	-0.328709	-0.477425	0.634131	female

Metabolites	logFC	t	P.Value	Sex
Methionine	0.124301	0.337433	0.736518	female
Methylamine	0.374658	1.111722	0.268999	female
Orn	-0.129983	-0.27592	0.783195	female
PC.aa.C24.0	-0.156676	-1.101696	0.273313	female
PC.aa.C28.1	0.257434	1.214795	0.227386	female
PC.aa.C30.0	-0.272838	-1.211572	0.228612	female
PC.aa.C32.0	-0.171127	-0.828824	0.409234	female
PC.aa.C32.1	-0.391651	-0.93831	0.35041	female
PC.aa.C32.2	-0.405601	-1.615362	0.109472	female
PC.aa.C32.3	-0.178055	-0.811932	0.418814	female
PC.aa.C34.1	0.022586	0.044673	0.964459	female
PC.aa.C34.2	0.140306	0.257062	0.797674	female
PC.aa.C34.3	0.074603	0.238352	0.812109	female
PC.aa.C34.4	0.528102	2.447999	0.016158	female
PC.aa.C36.0	0.046533	0.268997	0.788502	female
PC.aa.C36.1	0.070749	0.163143	0.870744	female
PC.aa.C36.2	-0.28417	-0.512148	0.609709	female
PC.aa.C36.3	-0.005494	-0.010354	0.99176	female
PC.aa.C36.4	0.489069	0.896564	0.372166	female
PC.aa.C36.5	0.088429	0.29348	0.76978	female
PC.aa.C36.6	-0.12813	-0.575553	0.566247	female
PC.aa.C38.0	0.288919	1.3222	0.189203	female
PC.aa.C38.3	0.205655	0.711647	0.478387	female
PC.aa.C38.4	0.895398	2.872704	0.004997	female
PC.aa.C38.5	0.68424	1.907594	0.059394	female
PC.aa.C38.6	-0.232274	-0.789482	0.431751	female
PC.aa.C40.1	0.079227	0.513069	0.609066	female
PC.aa.C40.2	-0.4117	-1.279002	0.20394	female
PC.aa.C40.3	0.777666	3.134911	0.002273	female
PC.aa.C40.4	0.532357	2.23487	0.027715	female
PC.aa.C40.5	0.568741	2.400727	0.018265	female
PC.aa.C40.6	0.076455	0.334861	0.738452	female
PC.aa.C42.0	-0.157476	-1.0964	0.275612	female
PC.aa.C42.1	0.420999	2.54944	0.012353	female
PC.aa.C42.2	-0.072715	-0.425808	0.671189	female
PC.aa.C42.4	0.222075	0.960815	0.339028	female
PC.aa.C42.5	0.162043	0.661739	0.509703	female
PC.aa.C42.6	0.143264	0.441131	0.660097	female
PC.ae.C30.0	-0.118056	-0.629717	0.530357	female
PC.ae.C30.1	-0.248441	-0.992565	0.323386	female
PC.ae.C30.2	0.351402	1.930338	0.056479	female
PC.ae.C32.1	-0.153205	-0.53546	0.593554	female

Metabolites	logFC	t	P.Value	Sex
PC.ae.C32.2	0.236151	1.156781	0.250196	female
PC.ae.C34.0	0.009726	0.040155	0.968052	female
PC.ae.C34.1	-0.339326	-1.122968	0.264216	female
PC.ae.C34.2	0.024415	0.067067	0.946666	female
PC.ae.C34.3	0.419744	1.613999	0.109768	female
PC.ae.C36.0	0.051395	0.246846	0.805548	female
PC.ae.C36.1	-0.128291	-0.721893	0.472093	female
PC.ae.C36.2	-0.205579	-0.934636	0.352291	female
PC.ae.C36.3	-0.012449	-0.067155	0.946596	female
PC.ae.C36.4	0.692739	2.965434	0.003803	female
PC.ae.C36.5	0.427029	2.230759	0.027995	female
PC.ae.C38.0	0.010557	0.06614	0.947402	female
PC.ae.C38.1	0.102011	0.532567	0.595548	female
PC.ae.C38.2	-0.345931	-0.968295	0.335299	female
PC.ae.C38.3	0.264513	1.275744	0.205085	female
PC.ae.C38.4	0.576662	2.519064	0.013397	female
PC.ae.C38.5	0.27818	1.38486	0.169264	female
PC.ae.C38.6	0.427825	1.546994	0.125113	female
PC.ae.C40.1	0.082315	0.399446	0.69044	female
PC.ae.C40.2	0.820582	3.620418	0.000469	female
PC.ae.C40.3	0.405837	1.722407	0.088175	female
PC.ae.C40.4	0.143109	0.732741	0.465479	female
PC.ae.C40.5	0.27524	1.120708	0.265172	female
PC.ae.C40.6	-0.393389	-1.730012	0.086801	female
PC.ae.C42.0	0.02571	0.166433	0.868162	female
PC.ae.C42.1	-0.322898	-2.013253	0.046852	female
PC.ae.C42.2	0.094443	0.435258	0.66434	female
PC.ae.C42.3	-0.336008	-1.73608	0.085717	female
PC.ae.C42.5	-0.056784	-0.362088	0.718072	female
PC.ae.C44.3	0.094811	0.498331	0.619376	female
PC.ae.C44.4	-0.387348	-2.398969	0.018348	female
PC.ae.C44.5	-0.157679	-0.843248	0.401159	female
PC.ae.C44.6	-0.08418	-0.562181	0.575286	female
Phe	-0.520389	-1.173338	0.243526	female
Phenylacetate	-0.22022	-0.727142	0.468886	female
Pro	0.158894	0.346112	0.730006	female
Putrescine	-1.106691	-0.977228	0.330881	female
Pyruvic.acid	0.136692	0.311562	0.756041	female
Sarcosine	-0.286679	-0.659775	0.510958	female
SDMA	0.693624	1.178068	0.241644	female
Ser	0.023649	0.070205	0.944174	female
Serotonin	0.715262	1.583505	0.116554	female

Metabolites	logFC	t	P.Value	Sex
SM..OH..C14.1	-0.090148	-0.244454	0.807395	female
SM..OH..C16.1	-0.139564	-0.296422	0.76754	female
SM..OH..C22.1	-0.528018	-1.248509	0.214842	female
SM..OH..C22.2	0.203358	0.480666	0.631834	female
SM..OH..C24.1	0.378071	1.008889	0.315532	female
SM.C16.0	0.010116	0.152679	0.878968	female
SM.C16.1	-0.116334	-0.517936	0.605679	female
SM.C18.0	-0.131968	-0.23904	0.811578	female
SM.C18.1	0.212984	0.507366	0.613047	female
SM.C20.2	-0.107181	-0.482029	0.630869	female
SM.C24.0	0.133194	0.476526	0.634769	female
SM.C26.0	0.135458	0.49403	0.622399	female
Spermidine	-0.460588	-0.599279	0.550381	female
Succinate	0.769606	1.122917	0.264237	female
t4.OH.Pro	0.100324	0.168177	0.866794	female
Taurine	-0.981325	-1.176811	0.242144	female
Thr	-0.093279	-0.38498	0.701093	female
Trimethylamine	0.313573	0.695435	0.488441	female
Trp	-0.233424	-0.741471	0.460195	female
Tyr	-1.060479	-3.028108	0.003151	female
Tyrosine	-0.097005	-0.328808	0.743008	female
Uracil	0.370079	1.158295	0.24958	female
Valine	0.630444	1.08039	0.282641	female
X3.Hydroxyisovaleric.acid	-0.071235	-0.134887	0.89298	female
Xanthine	-0.445517	-1.047653	0.297396	female
Ac.Orn	-0.160472	-0.271786	0.790879	male
Acetic.acid	-0.170265	-0.305114	0.766041	male
Acetone	0.197433	0.385045	0.70763	male
Ala	-2.170013	-2.042405	0.06613	male
alpha.AAA	1.006931	0.846222	0.415666	male
Asp	-0.153968	-0.232397	0.820546	male
Aspartate	1.989682	3.306076	0.007109	male
Betaine	-1.413019	-1.973713	0.074368	male
Butyrate	-0.065761	-0.099818	0.922305	male
C0	0.503967	0.562588	0.585129	male
C10.1	0.414738	0.859551	0.40859	male
C10.2	-0.971957	-1.256808	0.235155	male
C12	0.381938	0.78273	0.450499	male
C12.1	0.049454	0.10082	0.921529	male
C12.DC	-0.556184	-1.406029	0.187647	male
C14	-0.451823	-0.772674	0.456185	male
C14.1	0.111547	0.146162	0.886468	male

**Metabolites**	**logFC**	**t**	**P.Value**	**Sex**
C14.1.OH	0.385547	0.540802	0.599549	male
C14.2	0.610795	1.456536	0.173511	male
C14.2.OH	-0.260123	-0.582346	0.572212	male
C16	-0.567946	-0.738871	0.475635	male
C16.1	-0.326011	-0.366879	0.72075	male
C16.1.OH	0.227692	0.221454	0.828844	male
C16.2	-0.534007	-0.921717	0.376677	male
C16.2.OH	0.139208	0.223107	0.827589	male
C16.OH	0.018426	0.014809	0.988453	male
C18	-0.278435	-0.267228	0.794295	male
C18.1	-1.198953	-1.112745	0.289817	male
C18.1.OH	-0.730267	-0.667671	0.518265	male
C18.2	-0.414249	-0.384956	0.707695	male
C2	-0.290121	-0.56719	0.582107	male
C3.DC..C4.OH.	0.817924	0.924625	0.375228	male
C3.OH	0.277303	0.533117	0.604679	male
C4.1	0.038982	0.094501	0.92643	male
C5	-0.072966	-0.182617	0.858459	male
C5.DC..C6.OH.	0.420775	0.636244	0.537781	male
C5.M.DC	-0.129979	-0.253863	0.804338	male
C5.OH..C3.DC.M.	0.442127	0.817009	0.431463	male
C7.DC	0.583806	0.926533	0.37428	male
C9	0.301945	0.712533	0.491144	male
Choline	-0.000686	-0.001234	0.999038	male
Cit	0.547538	0.810943	0.434792	male
Creatine	-0.535832	-0.744936	0.472107	male
Creatinine	-1.409707	-1.273592	0.229363	male
D.Galactose	0.422791	0.653349	0.527107	male
D.Glucose	0.914873	0.817711	0.431078	male
Dimethylamine	-0.154713	-0.233107	0.820009	male
Ethanol	-0.82823	-0.844046	0.416829	male
Formate	-0.256549	-0.391678	0.702864	male
Fumaric.acid	1.67499	2.20094	0.05028	male
Gln	0.702583	1.641518	0.129271	male
Glu	-0.946891	-2.107794	0.059091	male
Gly	0.336635	0.489556	0.634176	male
Glycerol	-2.804613	-2.145097	0.0554	male
Glycine	0.867902	1.693654	0.11875	male
His	-0.581329	-0.691372	0.503827	male
Hypoxanthine	0.152918	0.284563	0.781326	male
Isobutyric.acid	0.19425	0.135224	0.894906	male
Isoleucine	1.18922	1.671356	0.123153	male

**Metabolites**	**logFC**	**t**	**P.Value**	**Sex**
Isopropyl.alcohol	0.294346	0.557301	0.588611	male
Isovaleric.acid	1.177389	1.295888	0.22185	male
L.Fucose	-1.425451	-2.649309	0.022816	male
L.Glutamic.acid	0.79102	1.994619	0.071764	male
L.Lactic.acid	0.074767	0.11763	0.908506	male
L.Leucine	-0.041367	-0.049194	0.961657	male
L.Lysine	0.211099	0.487493	0.63559	male
L.Phenylalanine	1.04353	1.602327	0.137715	male
lysoPC.a.C14.0	-0.523022	-0.828903	0.424984	male
lysoPC.a.C16.0	-0.018758	-0.02963	0.976899	male
lysoPC.a.C16.1	0.41182	0.990648	0.343393	male
lysoPC.a.C17.0	0.215404	0.435335	0.671836	male
lysoPC.a.C18.0	-0.406463	-0.460473	0.65425	male
lysoPC.a.C18.1	-0.199041	-0.285418	0.780688	male
lysoPC.a.C18.2	-0.650327	-1.002913	0.337703	male
lysoPC.a.C20.4	-0.695416	-1.481151	0.166957	male
lysoPC.a.C24.0	0.721518	0.898722	0.388272	male
lysoPC.a.C26.0	-0.018673	-0.036607	0.971462	male
lysoPC.a.C26.1	-0.36665	-0.783358	0.450145	male
lysoPC.a.C28.0	0.095009	0.23529	0.818357	male
lysoPC.a.C28.1	0.057576	0.137007	0.893529	male
Met.SO	0.045033	0.079387	0.938167	male
Methanol	-1.681442	-1.408332	0.186982	male
Methionine	1.227243	2.645546	0.022969	male
Methylamine	-0.226414	-0.331907	0.74627	male
Orn	-0.483941	-0.604782	0.557735	male
PC.aa.C24.0	-0.479052	-0.758083	0.464517	male
PC.aa.C28.1	-0.416433	-0.976106	0.350231	male
PC.aa.C30.0	0.257336	0.395032	0.700459	male
PC.aa.C32.0	0.000419	0.000597	0.999534	male
PC.aa.C32.1	-0.177347	-0.316193	0.757844	male
PC.aa.C32.2	0.045839	0.120729	0.906108	male
PC.aa.C32.3	0.175521	0.470697	0.647159	male
PC.aa.C34.1	0.097601	0.101827	0.920748	male
PC.aa.C34.2	-0.669191	-0.838414	0.41985	male
PC.aa.C34.3	-0.049234	-0.092947	0.927636	male
PC.aa.C34.4	-0.392342	-0.960111	0.357866	male
PC.aa.C36.0	0.820657	1.386474	0.193375	male
PC.aa.C36.1	1.351277	2.089663	0.060969	male
PC.aa.C36.2	1.364345	1.929828	0.080122	male
PC.aa.C36.3	1.085777	1.29622	0.22174	male
PC.aa.C36.4	-0.880604	-1.076609	0.304965	male

**Metabolites**	**logFC**	**t**	**P.Value**	**Sex**
PC.aa.C36.5	-0.167732	-0.35546	0.729046	male
PC.aa.C36.6	0.05197	0.09863	0.923227	male
PC.aa.C38.0	0.699759	1.845368	0.092371	male
PC.aa.C38.3	-0.300922	-0.634282	0.539014	male
PC.aa.C38.4	-0.49801	-0.858852	0.408959	male
PC.aa.C38.5	-1.835346	-3.662195	0.003812	male
PC.aa.C38.6	-0.67955	-1.205063	0.253754	male
PC.aa.C40.1	0.194113	0.511317	0.619353	male
PC.aa.C40.2	0.819896	1.46516	0.17119	male
PC.aa.C40.3	-1.317356	-1.782068	0.102652	male
PC.aa.C40.4	-1.075785	-2.020927	0.068609	male
PC.aa.C40.5	0.075264	0.141633	0.88996	male
PC.aa.C40.6	-0.223066	-0.611927	0.553167	male
PC.aa.C42.0	0.009595	0.020569	0.983962	male
PC.aa.C42.1	-0.409874	-0.908279	0.383423	male
PC.aa.C42.2	0.183293	0.482177	0.639241	male
PC.aa.C42.4	-0.258776	-0.611378	0.553518	male
PC.aa.C42.5	-0.023822	-0.035586	0.972257	male
PC.aa.C42.6	0.178845	0.390444	0.70375	male
PC.ae.C30.0	-0.396348	-1.068945	0.308254	male
PC.ae.C30.1	-0.743403	-1.0486	0.317114	male
PC.ae.C30.2	-0.3789	-0.884094	0.395776	male
PC.ae.C32.1	0.342085	0.594236	0.564515	male
PC.ae.C32.2	0.019272	0.035881	0.972027	male
PC.ae.C34.0	0.937929	2.265164	0.044952	male
PC.ae.C34.1	0.823162	1.886853	0.086154	male
PC.ae.C34.2	1.11113	1.913188	0.082409	male
PC.ae.C34.3	0.135183	0.219862	0.830052	male
PC.ae.C36.0	0.779607	1.12494	0.284837	male
PC.ae.C36.1	0.466849	0.888467	0.393522	male
PC.ae.C36.2	0.496788	0.942907	0.36621	male
PC.ae.C36.3	-0.112632	-0.196663	0.847719	male
PC.ae.C36.4	0.25175	0.296509	0.772429	male
PC.ae.C36.5	-0.830351	-1.413858	0.185394	male
PC.ae.C38.0	0.039016	0.089945	0.929967	male
PC.ae.C38.1	0.191393	0.430338	0.675356	male
PC.ae.C38.2	0.573034	1.144683	0.276916	male
PC.ae.C38.3	-0.29298	-0.669919	0.516886	male
PC.ae.C38.4	-0.570204	-0.840557	0.418699	male
PC.ae.C38.5	-0.99682	-1.403451	0.188394	male
PC.ae.C38.6	-1.623155	-2.042018	0.066174	male
PC.ae.C40.1	-1.216257	-2.093721	0.060544	male

**Metabolites**	**logFC**	**t**	**P.Value**	**Sex**
PC.ae.C40.2	-0.787729	-1.428886	0.181133	male
PC.ae.C40.3	-0.178132	-0.425944	0.678459	male
PC.ae.C40.4	0.072557	0.183701	0.857629	male
PC.ae.C40.5	0.352636	0.480965	0.640075	male
PC.ae.C40.6	0.531309	1.201774	0.254975	male
PC.ae.C42.0	-0.134605	-0.3543	0.72989	male
PC.ae.C42.1	-0.199999	-0.507332	0.622055	male
PC.ae.C42.2	1.025084	1.929661	0.080144	male
PC.ae.C42.3	0.636167	1.084039	0.301802	male
PC.ae.C42.5	-0.111875	-0.256113	0.802645	male
PC.ae.C44.3	0.025139	0.063996	0.950135	male
PC.ae.C44.4	-0.036681	-0.093452	0.927245	male
PC.ae.C44.5	-0.28276	-0.691985	0.503458	male
PC.ae.C44.6	0.026364	0.067123	0.947702	male
Phe	-0.707254	-0.85472	0.411145	male
Phenylacetate	1.081284	1.822422	0.095984	male
Pro	-0.639248	-1.435737	0.179218	male
Putrescine	-0.170899	-0.173435	0.865497	male
Pyruvic.acid	0.975064	1.671925	0.123039	male
Sarcosine	-0.487717	-1.107078	0.292153	male
SDMA	0.330458	0.251299	0.806269	male
Ser	0.400708	0.929415	0.37285	male
Serotonin	-0.603207	-0.771941	0.456601	male
SM..OH..C14.1	0.461549	0.622581	0.546396	male
SM..OH..C16.1	-0.040873	-0.062002	0.951686	male
SM..OH..C22.1	0.68385	0.755839	0.465807	male
SM..OH..C22.2	0.33785	0.497716	0.628597	male
SM..OH..C24.1	1.247085	1.738352	0.110348	male
SM.C16.0	0.976324	0.993916	0.34187	male
SM.C16.1	0.170392	0.367051	0.720625	male
SM.C18.0	1.255731	1.851096	0.091489	male
SM.C18.1	0.037868	0.041647	0.967535	male
SM.C20.2	-0.510793	-1.221748	0.247633	male
SM.C24.0	-0.254028	-0.446995	0.663653	male
SM.C26.0	0.206897	0.274265	0.789022	male
Spermidine	-1.460411	-1.39247	0.191603	male
Succinate	-0.110859	-0.101791	0.920776	male
t4.OH.Pro	0.638881	0.68123	0.509976	male
Taurine	-0.331666	-0.353382	0.730559	male
Thr	0.197163	0.561009	0.586168	male
Trimethylamine	-0.479715	-0.450298	0.661343	male
Trp	-0.119849	-0.238376	0.816023	male

**Metabolites**	**logFC**	**t**	**P.Value**	**Sex**
Tyr	0.476201	1.067165	0.309021	male
Tyrosine	1.297501	1.767337	0.105189	male
Uracil	0.743289	0.742312	0.473632	male
Valine	0.423319	0.742619	0.473453	male
X3.Hydroxyisovaleric.acid	0.135492	0.198623	0.846223	male
Xanthine	-0.044541	-0.090346	0.929655	male

Table S4. Log-scaled mean concentration ratio of serum metabolites, along with results from sex-stratified linear models comparing metabolic profile differences for the pairwise comparison of BL vs. 12M_T3.

**Metabolites**	**logFC**	**t**	**P.Value**	**Sex**
Acetic.acid	0.275773	0.897095	0.371836	female
Acetoacetate	0.643823	1.445744	0.151395	female
Acetone	0.527329	1.600766	0.112603	female
ADMA	-0.067649	-0.230625	0.81808	female
alpha.AAA	-0.712137	-1.912426	0.058701	female
Asp	-0.112818	-0.730168	0.467005	female
Betaine	-0.076027	-0.321426	0.748563	female
C0	-0.144907	-0.994996	0.322155	female
C10	-0.361124	-1.215407	0.22709	female
C14.1	0.002053	0.011416	0.990914	female
C16	-0.073239	-0.586534	0.558846	female
C18	0.207993	0.24196	0.80931	female
C18.1	0.261896	1.832183	0.069919	female
C18.2	0.069727	0.418656	0.676371	female
C2	-0.29974	-1.365185	0.175277	female
C3	-0.208628	-1.008282	0.315767	female
C3.DC..C4.OH.	0.267589	1.127829	0.262107	female
C4	-0.178306	-0.874508	0.38395	female
C5	-0.023966	-0.100567	0.920097	female
Carnitine	-0.076349	-0.303003	0.762521	female
Choline	-0.130688	-0.419854	0.675498	female
Cit	0.123796	0.726756	0.469084	female
Citric.acid	0.45667	2.602471	0.010671	female
Creatine	-0.658619	-2.201805	0.029992	female
Creatinine	-0.011675	-0.054223	0.956866	female
D.Glucose	0.054452	0.2435	0.80812	female
Dimethyl.sulfone	-0.040704	-0.117025	0.907076	female
Ethanol	-0.328485	-1.19765	0.233902	female
Formate	-0.10863	-0.443874	0.658099	female
Glu	-0.238874	-1.073868	0.285482	female
Gly	0.147854	0.805486	0.422462	female
Glycerol	-0.066996	-0.340122	0.734483	female
H1	-0.080043	-0.948309	0.345272	female
Hypoxanthine	-0.084636	-0.240503	0.810436	female
Isobutyric.acid	-0.145689	-0.521876	0.602918	female
Isoleucine	-0.061394	-0.259921	0.795462	female
Isopropyl.alcohol	-0.193625	-0.589868	0.556618	female
Kynurenine	0.122259	0.722636	0.471601	female
L.Alanine	-0.023784	-0.095684	0.923964	female
L.Arginine	0.408717	1.291857	0.199403	female

Metabolites	logFC	t	P.Value	Sex
L.Histidine	-0.084311	-0.249315	0.803631	female
L.Lactic.acid	0.023641	0.108351	0.913936	female
Leu	-0.162166	-1.180322	0.24069	female
Lys	0.042747	0.361933	0.71817	female
lysoPC.a.C16.0	-0.044401	-0.335764	0.737756	female
lysoPC.a.C16.1	-0.004691	-0.028598	0.977243	female
lysoPC.a.C17.0	0.188532	1.374829	0.172276	female
lysoPC.a.C18.0	-0.071639	-0.548378	0.584662	female
lysoPC.a.C18.1	0.101986	0.629473	0.530484	female
lysoPC.a.C18.2	0.133767	0.753746	0.452784	female
lysoPC.a.C20.3	-0.032142	-0.183617	0.854688	female
lysoPC.a.C20.4	0.115818	0.67116	0.503675	female
lysoPC.a.C28.1	-0.234927	-1.342919	0.182357	female
Malonate	0.181031	0.553555	0.581127	female
Met	0.028032	0.166035	0.868467	female
Met.SO	0.03954	0.372148	0.710575	female
Orn	0.024168	0.136698	0.891546	female
PC.aa.C28.1	-0.024246	-0.243901	0.80781	female
PC.aa.C30.0	0.107474	0.79313	0.429591	female
PC.aa.C32.0	0.163331	1.625779	0.107164	female
PC.aa.C32.1	-0.103543	-0.516807	0.606439	female
PC.aa.C32.2	-0.372899	-1.922608	0.057392	female
PC.aa.C32.3	-0.037407	-0.318992	0.750402	female
PC.aa.C34.1	-0.064188	-0.459371	0.646971	female
PC.aa.C34.2	-0.087273	-0.800819	0.425147	female
PC.aa.C34.3	-0.202424	-1.532804	0.128501	female
PC.aa.C34.4	-0.486784	-2.987797	0.00354	female
PC.aa.C36.0	0.047424	0.167787	0.867092	female
PC.aa.C36.1	-0.178717	-1.259223	0.210899	female
PC.aa.C36.2	-0.209082	-1.525737	0.130252	female
PC.aa.C36.3	-0.210504	-1.631024	0.10605	female
PC.aa.C36.4	-0.034154	-0.26743	0.789693	female
PC.aa.C36.5	-0.221735	-1.311819	0.192603	female
PC.aa.C36.6	-0.603944	-2.848703	0.005336	female
PC.aa.C38.0	0.039036	0.314859	0.753529	female
PC.aa.C38.3	0.05779	0.346317	0.729837	female
PC.aa.C38.4	-0.150567	-1.29474	0.19841	female
PC.aa.C38.5	-0.11322	-0.892869	0.374084	female
PC.aa.C38.6	-0.121202	-0.890821	0.375177	female
PC.aa.C40.1	0.273128	2.701995	0.008106	female
PC.aa.C40.4	0.041059	0.252786	0.800956	female
PC.aa.C40.5	0.029482	0.177984	0.859098	female

Metabolites	logFC	t	P.Value	Sex
PC.aa.C40.6	-0.203368	-1.318891	0.190237	female
PC.aa.C42.0	0.091056	0.661522	0.509809	female
PC.aa.C42.1	0.084617	0.617025	0.538629	female
PC.aa.C42.5	0.155061	1.162524	0.247807	female
PC.aa.C42.6	0.028873	0.218013	0.827866	female
PC.ae.C30.0	-0.093938	-0.632019	0.528826	female
PC.ae.C30.1	0.102298	0.472887	0.637331	female
PC.ae.C32.1	0.303231	2.958222	0.003867	female
PC.ae.C32.2	0.123123	1.297316	0.197526	female
PC.ae.C34.0	0.084585	0.869139	0.386866	female
PC.ae.C34.1	0.134332	1.531226	0.128891	female
PC.ae.C34.2	0.114715	1.133369	0.259786	female
PC.ae.C34.3	0.281553	2.536176	0.012764	female
PC.ae.C36.0	0.052294	0.538077	0.591727	female
PC.ae.C36.1	-0.037856	-0.439674	0.661128	female
PC.ae.C36.2	0.040777	0.397557	0.691809	female
PC.ae.C36.3	0.051469	0.504213	0.615227	female
PC.ae.C36.4	-0.027798	-0.261818	0.794004	female
PC.ae.C36.5	0.134479	1.067773	0.28821	female
PC.ae.C38.0	0.1255	0.831655	0.407597	female
PC.ae.C38.1	-0.752902	-2.375496	0.019444	female
PC.ae.C38.2	0.120551	0.71932	0.473632	female
PC.ae.C38.3	0.014827	0.092134	0.926777	female
PC.ae.C38.4	-0.095683	-0.872446	0.385068	female
PC.ae.C38.5	-0.161367	-1.341906	0.182684	female
PC.ae.C38.6	-0.01531	-0.114758	0.908868	female
PC.ae.C40.1	0.032236	0.2779	0.781666	female
PC.ae.C40.2	0.098658	0.395703	0.693172	female
PC.ae.C40.3	0.157132	0.696107	0.487986	female
PC.ae.C40.4	0.1067	0.803209	0.423771	female
PC.ae.C40.5	0.040888	0.273081	0.785358	female
PC.ae.C40.6	-0.063783	-0.453467	0.651201	female
PC.ae.C42.1	0.02397	0.173237	0.862818	female
PC.ae.C42.2	0.047511	0.30568	0.760488	female
PC.ae.C42.3	-0.065045	-0.545873	0.586376	female
PC.ae.C42.4	0.194562	1.343435	0.182191	female
PC.ae.C42.5	0.078149	0.599059	0.550496	female
PC.ae.C44.3	0.029871	0.186811	0.852189	female
PC.ae.C44.4	0.030916	0.218183	0.827734	female
PC.ae.C44.5	0.062305	0.438968	0.661638	female
PC.ae.C44.6	0.093793	0.774964	0.440201	female
Pro	0.036237	0.264504	0.79194	female

Metabolites	logFC	t	P.Value	Sex
Propylene.glycol	0.400226	0.889052	0.376122	female
Putrescine	0.016786	0.082109	0.934725	female
Pyruvic.acid	-0.297336	-1.027074	0.306879	female
Sarcosine	-0.549115	-0.43336	0.665692	female
SDMA	0.004244	0.035876	0.971453	female
Serotonin	0.242394	0.440007	0.660888	female
SM..OH..C14.1	0.099372	0.957517	0.34063	female
SM..OH..C16.1	0.1408	1.167506	0.2458	female
SM..OH..C22.1	-0.326046	-3.014922	0.003263	female
SM..OH..C24.1	-0.103123	-0.930834	0.354195	female
SM.C18.0	0.00491	0.03931	0.968722	female
SM.C18.1	0.093256	0.721765	0.472134	female
SM.C20.2	-0.080089	-0.529172	0.597866	female
SM.C24.1	0.171038	1.352478	0.179292	female
SM.C26.0	0.034609	0.230986	0.817801	female
SM.C26.1	0.223724	1.300253	0.196522	female
Succinate	0.062863	0.402481	0.688195	female
t4.OH.Pro	0.362081	1.279208	0.203803	female
Taurine	-0.012294	-0.068184	0.945776	female
Thr	0.17805	1.429551	0.155982	female
Trp	0.006189	0.036077	0.971293	female
Tyr	-0.034053	-0.206667	0.836693	female
Urea	0.083495	0.259115	0.796083	female
Val	-0.294938	-2.047738	0.043223	female
X1.Methylhistidine	-0.194005	-1.117242	0.266585	female
X2.Hydroxybutyric.acid	0.256112	1.124009	0.263717	female
X3.Hydroxybutyric.acid	0.706203	1.627078	0.106887	female
Acetic.acid	-0.088497	-0.176749	0.863597	male
Acetoacetate	-0.147787	-0.248782	0.809081	male
Acetone	-0.06015	-0.138176	0.893126	male
ADMA	1.064197	1.176033	0.26958	male
alpha.AAA	0.150321	0.275301	0.789265	male
Asp	0.035277	0.111927	0.913323	male
Betaine	0.467354	1.989444	0.077679	male
C0	-0.040263	-0.125216	0.903089	male
C10	-0.501619	-0.966062	0.359109	male
C14.1	-0.096474	-0.197939	0.847466	male
C16	0.066214	0.284249	0.782614	male
C18	-0.133539	-0.469468	0.649836	male
C18.1	-0.52887	-1.841999	0.098403	male
C18.2	-0.312026	-1.071385	0.311725	male
C2	-0.412453	-1.36164	0.206231	male

Metabolites	logFC	t	P.Value	Sex
C3	0.783936	1.627974	0.137773	male
C3.DC..C4.OH.	-0.151785	-0.400833	0.697838	male
C4	0.526242	1.812431	0.10314	male
C5	0.536239	0.903097	0.389871	male
Carnitine	-0.612217	-2.756615	0.022116	male
Choline	-0.120771	-0.299118	0.771603	male
Cit	-0.040581	-0.19394	0.850505	male
Citric.acid	0.89997	2.392753	0.040221	male
Creatine	-0.073107	-0.197678	0.847664	male
Creatinine	-0.796763	-2.758418	0.022051	male
D.Glucose	-0.138163	-0.816187	0.435342	male
Dimethyl.sulfone	0.249457	0.683957	0.511128	male
Ethanol	0.946362	2.273386	0.048926	male
Formate	0.176693	0.59069	0.569179	male
Glu	0.245731	0.415485	0.687461	male
Gly	-0.704554	-2.175997	0.057375	male
Glycerol	0.319159	0.939515	0.371855	male
H1	0.045023	0.265968	0.796222	male
Hypoxanthine	0.57913	0.80992	0.438755	male
Isobutyric.acid	0.010655	0.036149	0.971948	male
Isoleucine	-0.630805	-1.698892	0.123363	male
Isopropyl.alcohol	0.725614	1.382788	0.199881	male
Kynurenine	0.46415	1.593229	0.145375	male
L.Alanine	-0.431953	-0.802236	0.442965	male
L.Arginine	0.552935	1.396022	0.195993	male
L.Histidine	-0.36247	-0.500784	0.628471	male
L.Lactic.acid	0.028356	0.248011	0.80966	male
Leu	0.257647	0.882058	0.400558	male
Lys	-0.037502	-0.124943	0.903299	male
lysoPC.a.C16.0	-0.405805	-1.810787	0.10341	male
lysoPC.a.C16.1	-0.27652	-1.03148	0.329083	male
lysoPC.a.C17.0	-0.494747	-1.968386	0.080363	male
lysoPC.a.C18.0	-0.290799	-1.438741	0.183884	male
lysoPC.a.C18.1	-0.0503	-0.264395	0.797396	male
lysoPC.a.C18.2	-0.134555	-0.341564	0.740474	male
lysoPC.a.C20.3	-0.721957	-2.064324	0.068814	male
lysoPC.a.C20.4	-0.491866	-1.732741	0.116982	male
lysoPC.a.C28.1	-0.292148	-0.94416	0.369602	male
Malonate	-0.319876	-0.72342	0.487691	male
Met	0.426005	1.096895	0.301005	male
Met.SO	1.917722	2.059107	0.069398	male
Orn	0.284288	1.42792	0.186888	male

Metabolites	logFC	t	P.Value	Sex
PC.aa.C28.1	-0.090213	-0.434392	0.674173	male
PC.aa.C30.0	0.541029	1.922151	0.086566	male
PC.aa.C32.0	0.538093	3.244038	0.010016	male
PC.aa.C32.1	0.325005	1.010052	0.338702	male
PC.aa.C32.2	0.03469	0.133011	0.897095	male
PC.aa.C32.3	-0.012999	-0.065843	0.948934	male
PC.aa.C34.1	0.263221	0.984091	0.350638	male
PC.aa.C34.2	-0.232103	-1.235626	0.247699	male
PC.aa.C34.3	0.088716	0.415737	0.687283	male
PC.aa.C34.4	-0.420628	-2.63068	0.027198	male
PC.aa.C36.0	-0.233998	-0.419466	0.684654	male
PC.aa.C36.1	0.150963	0.669771	0.519719	male
PC.aa.C36.2	0.045968	0.144553	0.888231	male
PC.aa.C36.3	-0.109122	-0.464516	0.653247	male
PC.aa.C36.4	-0.470867	-2.118811	0.062982	male
PC.aa.C36.5	-0.440837	-1.231247	0.249256	male
PC.aa.C36.6	-0.466134	-1.152885	0.278488	male
PC.aa.C38.0	-0.02307	-0.115281	0.910739	male
PC.aa.C38.3	-0.271521	-1.099579	0.299894	male
PC.aa.C38.4	-0.158317	-0.88035	0.401435	male
PC.aa.C38.5	-0.087249	-0.297845	0.772543	male
PC.aa.C38.6	-0.315276	-0.793395	0.447842	male
PC.aa.C40.1	-0.502645	-2.7151	0.023675	male
PC.aa.C40.4	0.049314	0.250145	0.808059	male
PC.aa.C40.5	-0.001282	-0.007437	0.994228	male
PC.aa.C40.6	-0.266593	-0.877184	0.403064	male
PC.aa.C42.0	-0.110749	-0.301694	0.769701	male
PC.aa.C42.1	-0.507861	-1.100393	0.299558	male
PC.aa.C42.5	0.033806	0.121805	0.905714	male
PC.aa.C42.6	-0.183722	-1.14359	0.282131	male
PC.ae.C30.0	0.384566	1.45797	0.178648	male
PC.ae.C30.1	-0.217104	-0.708424	0.496516	male
PC.ae.C32.1	0.228481	1.163926	0.27421	male
PC.ae.C32.2	0.239158	1.435703	0.184723	male
PC.ae.C34.0	0.214314	0.721294	0.488936	male
PC.ae.C34.1	0.250483	1.671289	0.128801	male
PC.ae.C34.2	0.167761	0.878995	0.402132	male
PC.ae.C34.3	0.153113	0.598628	0.564097	male
PC.ae.C36.0	0.233222	1.307997	0.223109	male
PC.ae.C36.1	0.014293	0.087457	0.932212	male
PC.ae.C36.2	0.224681	1.064512	0.314664	male
PC.ae.C36.3	-0.015184	-0.088365	0.931511	male

Metabolites	logFC	t	P.Value	Sex
PC.ae.C36.4	-0.241992	-1.345317	0.211248	male
PC.ae.C36.5	-0.121813	-0.491915	0.634487	male
PC.ae.C38.0	-0.637385	-1.877615	0.092967	male
PC.ae.C38.1	0.561475	1.194716	0.262558	male
PC.ae.C38.2	-0.262964	-0.848182	0.418197	male
PC.ae.C38.3	0.201007	0.770934	0.460392	male
PC.ae.C38.4	0.002636	0.012016	0.990674	male
PC.ae.C38.5	0.144313	0.57409	0.579888	male
PC.ae.C38.6	-0.641334	-1.774954	0.109449	male
PC.ae.C40.1	-0.316894	-2.073692	0.067775	male
PC.ae.C40.2	-0.458767	-0.854183	0.415034	male
PC.ae.C40.3	-0.426767	-0.780994	0.454742	male
PC.ae.C40.4	0.138007	0.673356	0.51754	male
PC.ae.C40.5	0.179429	0.832905	0.426324	male
PC.ae.C40.6	-0.190625	-0.766848	0.462699	male
PC.ae.C42.1	-0.238131	-0.527437	0.61057	male
PC.ae.C42.2	0.03031	0.10103	0.921729	male
PC.ae.C42.3	-0.148814	-0.825481	0.430313	male
PC.ae.C42.4	0.313521	0.65245	0.530327	male
PC.ae.C42.5	0.129742	0.442587	0.66845	male
PC.ae.C44.3	0.229884	0.858805	0.412609	male
PC.ae.C44.4	0.41601	1.270496	0.235585	male
PC.ae.C44.5	0.214191	0.500328	0.62878	male
PC.ae.C44.6	-0.039197	-0.122966	0.904821	male
Pro	-0.263767	-0.79141	0.448942	male
Propylene.glycol	0.979304	1.854204	0.096508	male
Putrescine	-0.579085	-1.476953	0.173607	male
Pyruvic.acid	-0.261548	-0.707094	0.497304	male
Sarcosine	0.750766	0.410254	0.691158	male
SDMA	-0.045437	-0.170795	0.868141	male
Serotonin	-1.566734	-1.72268	0.118846	male
SM..OH..C14.1	0.003214	0.016934	0.986857	male
SM..OH..C16.1	-0.139643	-0.673562	0.517415	male
SM..OH..C22.1	-0.322201	-1.86372	0.095054	male
SM..OH..C24.1	-0.421814	-2.46394	0.035779	male
SM.C18.0	0.062509	0.302286	0.769264	male
SM.C18.1	0.12288	0.708864	0.496256	male
SM.C20.2	0.458669	1.119335	0.291815	male
SM.C24.1	0.062778	0.297293	0.772951	male
SM.C26.0	-0.425767	-1.217566	0.254172	male
SM.C26.1	0.187077	0.645078	0.534881	male
Succinate	0.197721	0.584951	0.572869	male

**Metabolites**	**logFC**	**t**	**P.Value**	**Sex**
t4.OH.Pro	0.020662	0.054096	0.958034	male
Taurine	-0.215618	-0.866743	0.408467	male
Thr	0.073922	0.28578	0.781478	male
Trp	0.317926	1.458573	0.178486	male
Tyr	0.352914	1.246665	0.24381	male
Urea	-0.269257	-0.753194	0.470466	male
Val	0.409974	1.650424	0.133055	male
X1.Methylhistidine	-0.052255	-0.14383	0.888786	male
X2.Hydroxybutyric.acid	-0.087539	-0.354504	0.731079	male
X3.Hydroxybutyric.acid	0.506625	0.662926	0.523896	male

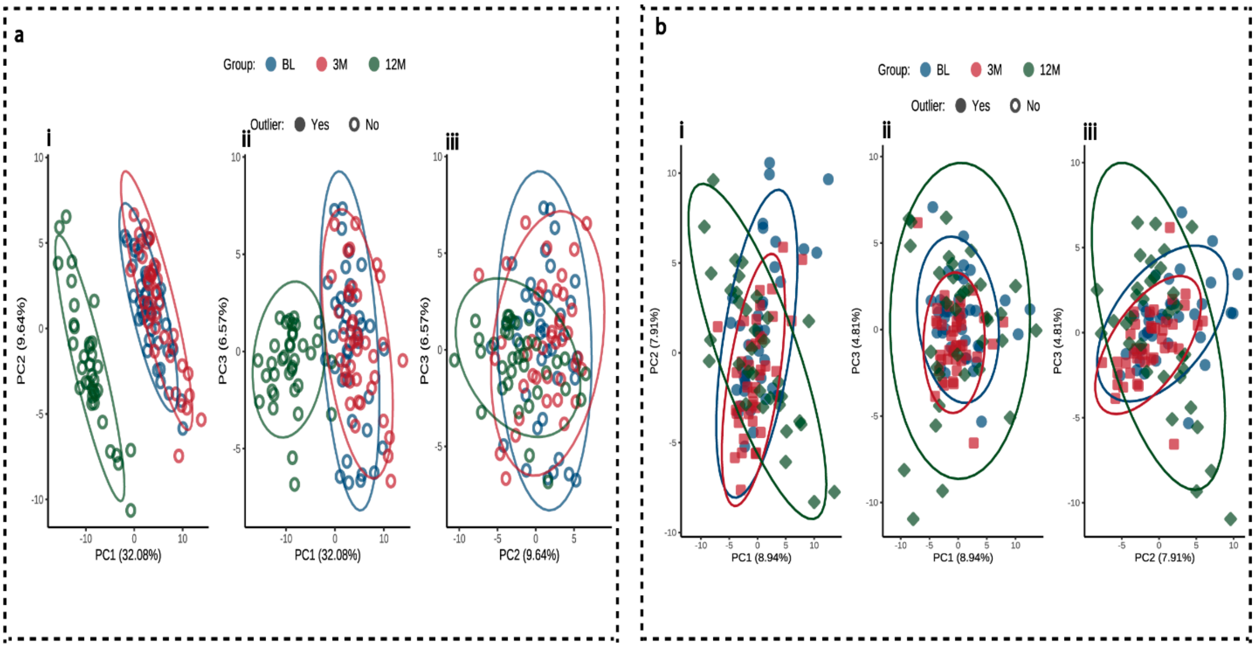

Figure S1. Quality-control assessment of longitudinally collected fecal metabolomics data using principal component analysis (PCA) to determine the existence of systematic variation, outliers, and potential batch effects (a) before and (b) after data preprocessing.

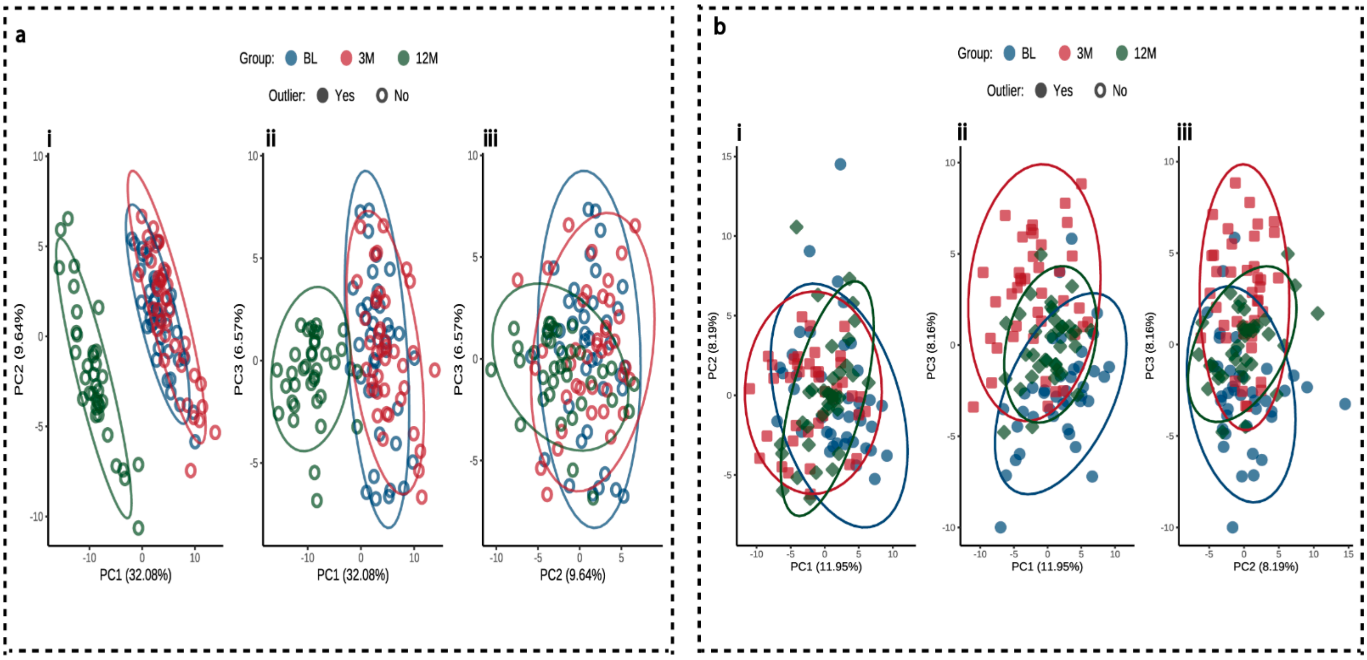

Figure S2. Quality-control assessment of longitudinally collected serum metabolomics data by Principal Component Analysis (PCA) to identify systematic variation, outliers and potential batch effects **(a)** before and **(b)** after proper data preprocessing.

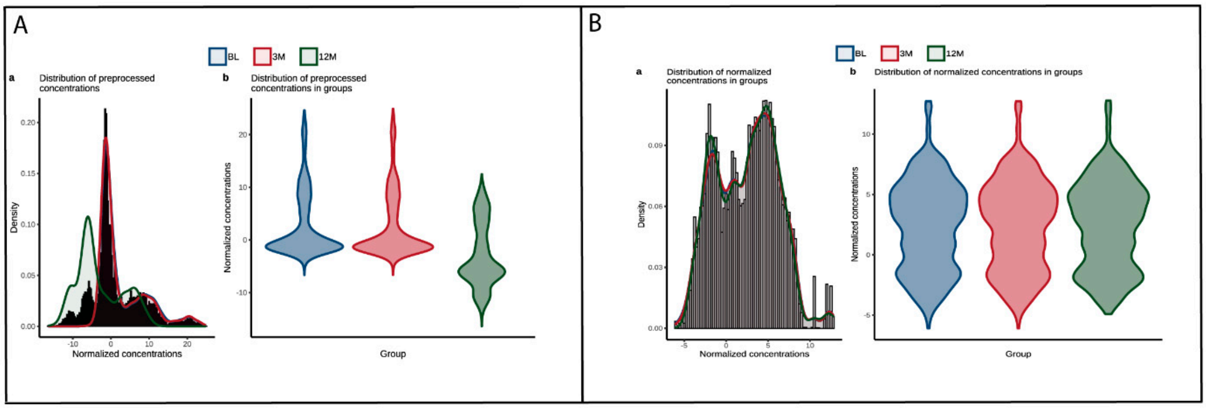

Figure S3. Histogram graphs displayed the overall signal intensity distribution across fecal metabolomic datasets before **(A)** and after **(B)** preprocessing, with aggregated conditions **(a)** and corresponding group-specific density plots of the normalized signal profiles **(b)**.

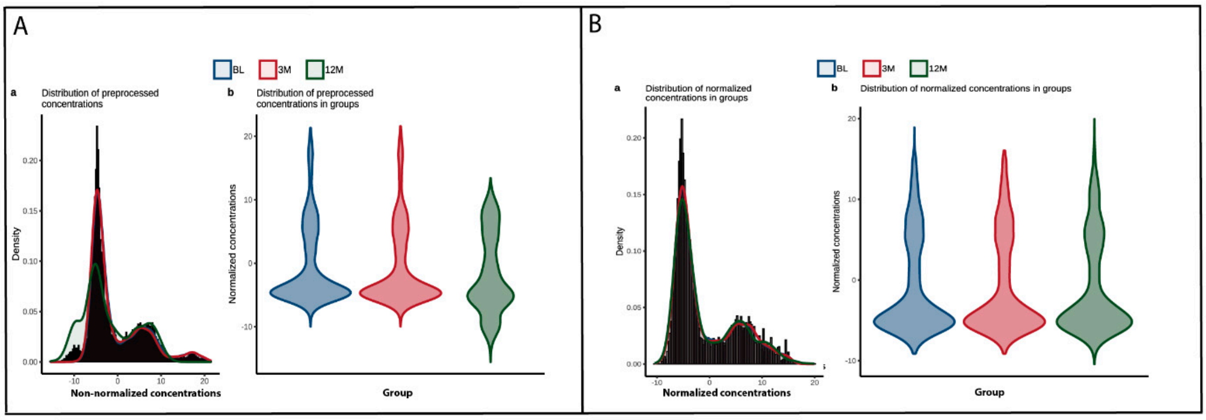

Figure S4. Histogram graphs displayed the overall signal intensity distribution across serum metabolomic datasets before **(A)** and after **(B)** preprocessing, with aggregated conditions **(a)** and corresponding group-specific density plots of the normalized signal profiles **(b)**.

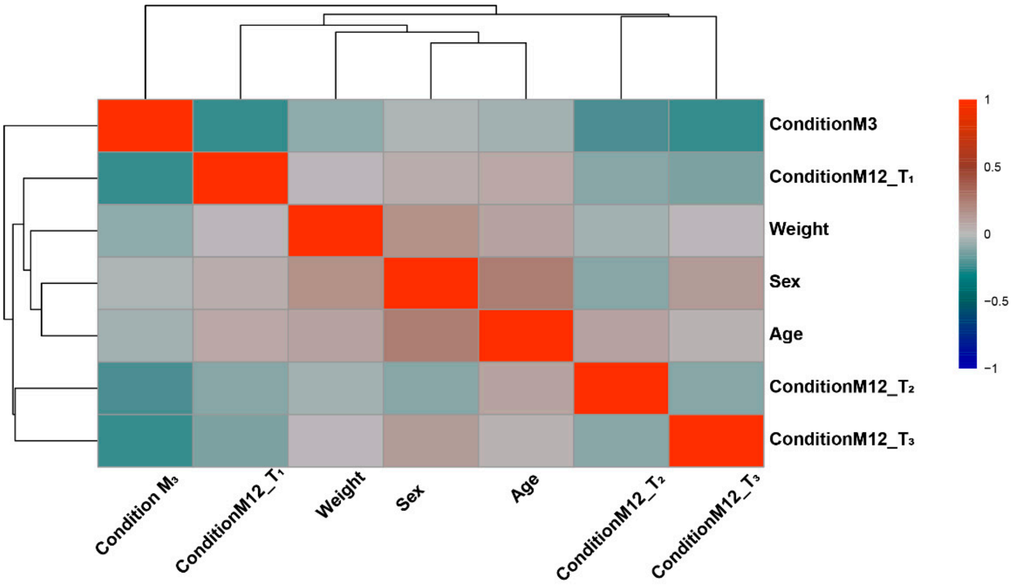

Figure S5. Hierarchical clustering and heatmap of the Pearson's correlation between all covariates incorporated into linear models based on fecal metabolomics data, so that covariates with higher correlations will be dropped from the reduced model.

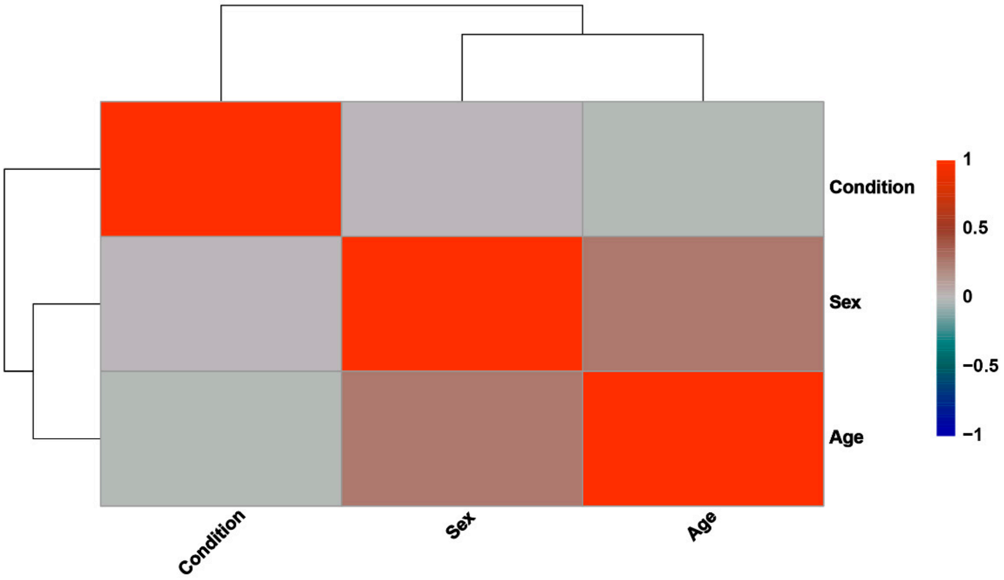

Figure S6. Hierarchical clustering and heatmap of the Pearson's correlation between all covariates incorporated into linear models based on serum metabolomics data, so that covariates with higher correlations will be dropped from the reduced model.

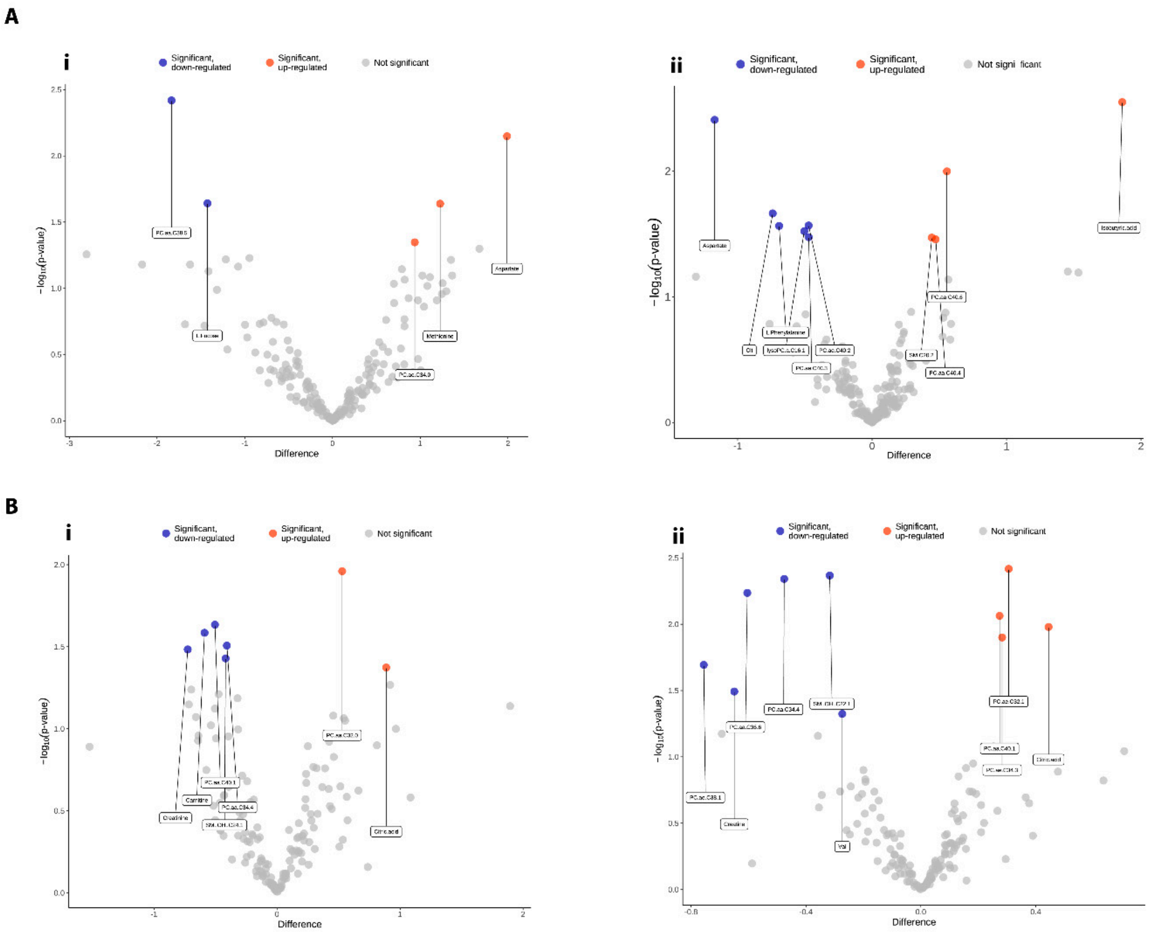

Figure S7. Volcano plots showing the results of sex-stratified analysis **(i-ii)** for both fecal **(A)** and serum **(B)** metabolomics data.

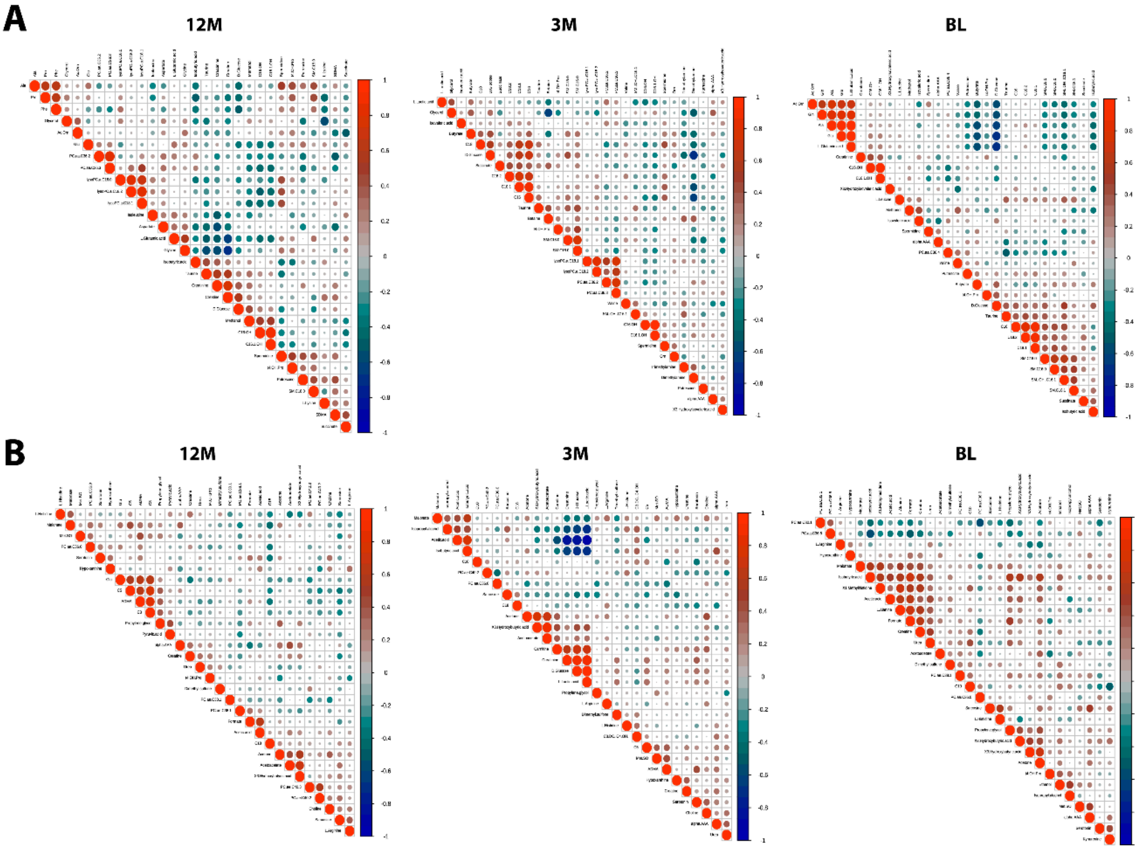

Figure S8. Correlations among the 30 most significantly perturbed metabolites for each time point (BL, 3M, 12M) for fecal **(A)** and serum **(B)** metabolomics data obtained from patients who underwent SG.
